# Decoding the molecular landscape: HER2 and PD-L1 in advanced gastric cancer

**DOI:** 10.3389/fimmu.2025.1567308

**Published:** 2025-05-30

**Authors:** Jun Yao, Qiang Sun, Han Wu, Xiaokai Zhao, Pengmin Yang, Xiaozhi Wang, Xintao Wang, Meiping Gu, Jieyi Li, Yuansi Zheng, Ziying Gong, Daoyun Zhang, Weijun Wang

**Affiliations:** ^1^ Department of Gastrointestinal Surgery, Changzheng Hospital, Naval Medical University, Shanghai, China; ^2^ Jiaxing Key Laboratory of Precision Medicine and Companion Diagnostics, Jiaxing Yunying Medical Inspection Co., Ltd., Jiaxing, China; ^3^ Department of Pathology, Zhejiang Cancer Hospital, Hangzhou, Zhejiang, China

**Keywords:** HER2 amplification, TP53 mutation, PD-L1 CPS strata, advanced gastric cancer, prognosis

## Abstract

**Introduction:**

Epidermal growth factor receptor 2 (*HER2*) and programmed cell death ligand 1 (PD-L1) are pivotal therapeutic targets for advanced gastric cancer (GC). Nevertheless, the correlation between them, along with the clinical and genomic characteristics and prognosis differences across distinct molecular subtypes, remains elusive.

**Methods:**

In this retrospective study, 390 advanced GC patients provided both tumor tissue and paired blood samples for Next-Generation Sequencing (NGS) of 639 tumor-related genes, along with PD-L1 immunohistochemical staining. *HER2* amplification was further validated using FISH in 254 patients. We analyzed the clinical and molecular characteristics of the subgroups based on *HER2* amplification and PD-L1 CPS scores.

**Results and discussion:**

The highest consistency with FISH for *HER2* amplification was observed when the positive threshold for NGS detection was set to 2.5. *TP53* mutation rate peaked at 59%, which was significantly higher in cases with *HER2* amplification (P<0.01). Patients with both *HER2* amplification and *TP53* mutations exhibited notably shorter survival rates than cases with only *TP53* mutations (P<0.05). Furthermore, *HER2* amplification did not correlate with PD-L1 expression. A stratified analysis of PD-L1 expression revealed distinct clinical and molecular features. When the CPS threshold is set at 5, 10, and 20, PD-L1 positive patients have a significantly higher proportion of high tumor mutational burden (TMB-H) and high microsatellite instability (MSI-H) status compared to PD-L1 negative patients. Additionally, patients with PD-L1 CPS ≥5 demonstrate an enrichment of mutations in key signaling pathways, such as PI3K, TGFβ, and Wnt/β-catenin.

**Conclusion:**

Overall, our study highlights the prognostic significance of *HER2* amplification and TP53 mutations in patients with advanced GC. Stratified analysis of PD-L1 expression may help to identify candidates for targeted immunotherapy in this patient population.

## Graphical Abstract

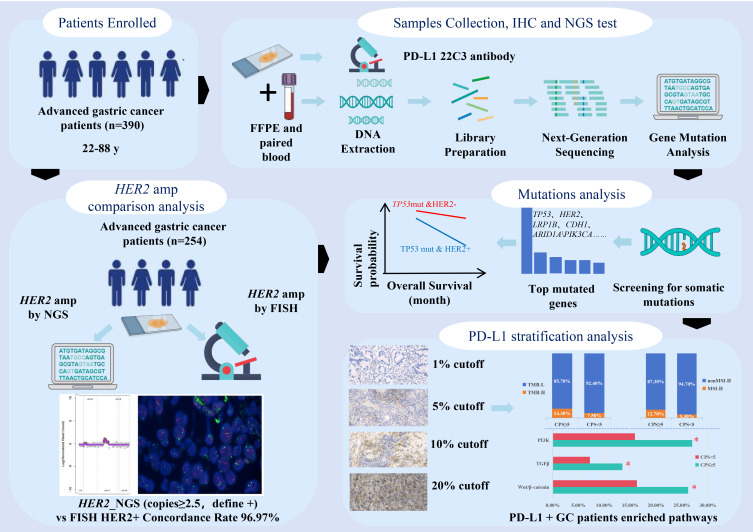

## Background

Gastric cancer (GC) is the fifth leading cause of cancer-related mortality worldwide (1). In recent years, substantial progress has been made in the advancement of molecularly targeted therapies, leading to optimized treatment regimens and enhanced overall survival (OS) outcomes in patients with advanced GC. Among the diverse molecular targets of GC, human epidermal growth factor receptor 2 (HER2, *ERBB2*) has emerged as a pivotal therapeutic target. In the Chinese population, approximately 12% of GC cases have been identified to exhibit *HER2* gene amplification (2). The landmark phase III ToGA trial demonstrated that adding trastuzumab to chemotherapy significantly improved survival in HER2-positive advanced G/GEJ adenocarcinoma patients (mOS 13.8 months vs. 11.1 months; HR=0.74, 95% CI 0.60–0.91; P = 0.0046), thereby establishing this regimen as the global first-line standard (3). The KEYNOTE-811 trial demonstrated that adding pembrolizumab to trastuzumab and chemotherapy significantly improved objective response rates (ORR 74.4% vs 51.9%) in HER2-positive gastric cancer (4). Based on these results, the 2023 ESMO Clinical Practice Guidelines (v2.0) now recommend PD-1 inhibitors combined with trastuzumab and chemotherapy as the preferred first-line regimen for HER2-positive metastatic disease (5), which has also been incorporated into the Pan-Asian adapted ESMO Clinical Practice Guidelines for standardized management of gastric cancer patients across Asian regions (6). Several randomized Phase III trials conducted in patients with HER2-positive gastric or gastroesophageal junction (G/GEJ) cancer have further validated the efficacy of HER2-targeted therapy. These trials included TRIO-013/LOGiC, which investigated the use of lapatinib in combination with oxaliplatin and capecitabine as a first-line treatment (7); TyTAN, which explored the use of paclitaxel plus lapatinib as a second-line treatment (8); and the GATSBY trial, which evaluated the efficacy of T-DM1 as a second-line treatment option (9).

Additionally, immune checkpoint inhibitors targeting the programmed death-1 (PD-1) and programmed death-ligand 1 (PD-L1) pathways have emerged as promising treatment options for advanced GCs. In PD-L1 combined positive score (CPS) ≥1 patients, pembrolizumab combined with trastuzumab/chemotherapy achieved superior OS versus chemotherapy/trastuzumab (20.1 months vs 15.7 months; HR=0.79, 95%CI 0.66-0.95, P=0.004) (10). Recent advances have led to the approval of nivolumab in combination with chemotherapy as a first-line treatment for HER2-negative advanced GCs, particularly those with positive PD-L1 expression (CPS ≥ 5), based on the results of the CheckMate-649 trial (11). Based on the landmark KEYNOTE-859 trial, the FDA has approved pembrolizumab for advanced gastric cancer patients with PD-L1 CPS ≥1, demonstrating significant survival benefits (mOS: 13.0 months vs 11.4 months; HR=0.74, 95%CI 0.65-0.84). This therapeutic advantage became more pronounced in the CPS ≥10 subgroup (mOS 15.7 months vs 11.8 months; HR=0.65, 95% CI 0.53-0.79) (12). In contrast, the KEYNOTE-590 trial for esophageal cancer established a CPS≥10 threshold for achieving significant clinical benefit (13). Notably, the RATIONALE-305 trial introduced a novel PD-L1 assessment metric-Tumor Area Positivity (TAP), which quantifies both tumor and immune cell staining. In TAP≥5% patients, tislelizumab+chemotherapy achieved superior OS (median OS: 17.2 vs 12.6 months; HR=0.74, 95% CI 0.59-0.94; P=0.006) (14). These studies indicate that PD-L1 is a crucial therapeutic target for patients with advanced or metastatic GC; however, the tumor heterogeneity underscores the necessity of assay-specific biomarker validation in GC immunotherapy.

Given the clinical relevance of *HER2* amplification and PD-L1 expression in GC, we conducted a retrospective analysis of genetic testing data to evaluate the concordance between next-generation sequencing (NGS) and fluorescence *in situ* hybridization (FISH), the gold standard for *HER2* amplification detection. By examining associated molecular profiles and clinical variations, we further stratified PD-L1 expression levels and investigated potential molecular distinctions across different subgroups. This study enhances our understanding of the molecular landscape of GC and provides valuable insights into the clinical implications of *HER2* and PD-L1 status. Our findings contribute to the advancement of personalized treatment strategies and molecularly targeted therapies in GC.

## Methods

### Patients and sample characteristics

From June 2021 to October 2023, 390 patients with pathologically diagnosed advanced GC were enrolled in the present study at the Changzheng Hospital. Each patient underwent a pathological diagnosis and was required to provide both tumor tissue and paired blood samples. Cancer diagnosis was initially established through clinical and X-ray findings, and later confirmed via histological analysis of tumor biopsies. All patients had not received systemic therapies including anti-HER2 treatment at the time of sample collection. Exclusion criteria for the study included cases where GC was not pathologically confirmed, cases where tissue or blood samples were not provided, and cases where the cell blocks of the samples contained less than 20% tumor cells. Clinical data, including information on age and sex, were retrieved from medical records. Written informed consent was obtained from all participants, and the study was approved by the institutional review board of our hospital (2023SL006).

### DNA extraction and library construction

Tumor DNA and blood genomic DNA were extracted using a human tissue DNA extraction kit (Shanghai YunYing) and a human blood genomic DNA extraction kit (Shanghai YunYing), respectively, according to the manufacturer’s protocols. DNA was eluted in elution buffer and its concentration and purity were evaluated using a NanoDrop spectrophotometer. DNA was stored at -20°C until use. Library preparation was performed using a VAHTS Universal DNA Library Prep Kit (Illumina). Target enrichment was performed using Shanghai YunYing’s optimized probes, which target exons and introns of 639 cancer-related genes. Sequencing was performed on an Illumina NextSeq500 platform using the manufacturer’s protocol with read lengths of 2 × 151 bp.

### Next-generation sequencing -based assay and bioinformatics analysis

Raw sequencing data quality control was performed using FastQC (v0.11.2) with default parameters. Adapter trimming and quality filtering were implemented through a custom Python script requiring: (1) Phred quality score ≥30 (Q30) across ≥90% of bases per read; (2) minimum retained read length of 75 bp after trimming; (3) exclusion of reads containing >5% ambiguous N bases. Processed reads were aligned to the GRCh37/hg19 reference genome using BWA-MEM (Burrows-Wheeler Aligner v0.7.17) with parameters: -p -K 100000 -Y -M -I 100,45,310,25, and read group information specified via -R “${tumor_read_group}”. Post-alignment processing included two sequential steps: (1) Duplicate sequences removal using Picard MarkDuplicates (v2.25.0) with VALIDATION_STRINGENCY=LENIENT and REMOVE_SEQUENCING_DUPLICATES=true; (2) The BAM files were then realigned and recalled using GATK4(v4.1.7.0), which was also used to detect mutations. For somatic variant detection, we employed a dual-caller strategy: (i) SNVs were initially called using GATK4 (v4.1.7.0) UnifiedGenotyper (-stand_call_conf 30, -stand_emit_conf 10, -mbq 20) followed by VarDict (v1.8.2) filtering (VarDictJava.jar -b refined.bam -G ${ref_fa} -th ${task.cpus} -N ${params.SampleId} -hotspot ${core_hotspots_vcf} -c 1 -S 2 -E 3 target.bed); (ii) insertion or deletion (indel) were identified using Pindel (v0.2.5b8) requiring ≥5 unique supporting reads and ≥10% allele frequency, with exclusion of variants in homopolymer regions >5bp. COSMIC v90 hotspot mutations were called with relaxed thresholds (–min-var-freq 0.01, –min-reads2 5).

Tumor-normal paired analysis was conducted through Mutect2 v4.5.0.2 (–interval-padding 20 –assembly-region-padding 50 –max-reads-per-alignment-start 500 –force-call-filtered-alleles true –genotype-germline-sites true –genotype-pon-sites true –max-mnp-distance 5 –max-num-haplotypes-in-population 255 –max-unpruned-variants 100 –pruning-lod-threshold 1.1 –kmer-size 13 –kmer-size 27 –kmer-size 73) and VarDict somatic pipeline. Variant annotation utilized ANNOVAR (2019Oct24) with SnpEff (v4.3) for functional prediction, ClinVar 20191105 for clinical interpretation, and COSMIC v90 for cancer association. Tumor mutational burden (TMB) was calculated as (total coding mutations)/1.36 Mb, including synonymous variants but excluding those with >0.1% frequency in gnomAD (v2.1). Microsatellite instability (MSI) status was determined using MSIsensor (v0.6) (15) analyzing ≥50 loci, with MSI-high defined as ≥40% unstable loci with parameters (msisensor msi -d./config/b37_microsatellites_singlebase_morethan10.list -t $i -e./config/b37_annotation.bed -o./MSISensor_result_background/${i##*/} -b 5 -p 10). We used 29 microsatellite sites as input files for the MSI detection of tumor-only patterns. MSI score was defined as the percentage of unstable microsatellites among the microsatellites used. Each microsatellite site had at least 20 spanning reads and single nucleotide mutations. Gene copy number gains were interpreted along with the amplicon sequencing data using the oncoCNV (v6.4) method, as described in a previous study (16). Analysis of gene structural variation was conducted with SvABA (v1.2.0) with parameters (–chunk-size 5000 –max-reads 80000 –max-coverage 5000 –max-reads-mate-region 2000).

### *HER2* amplification analysis by FISH method

We assessed *HER2* gene amplification status in 254 advanced GCs using FISH. Tumor tissues were processed from formalin-fixed paraffin-embedded (FFPE) samples using a commercially available detection kit (YunYing Co., Ltd., Shanghai, China). For *HER2* evaluation, dual-probe FISH was performed targeting both the *HER2* gene and chromosome 17 centromere (*CEP17*) as a reference. Samples were considered *HER2*-positive if they met both criteria: *HER2/CEP17* ratio ≥ 2.0, and average *HER2* copies/cells ≥ 4.0. All analyses were conducted by counting signals in ≥30 non-overlapping tumor cells under high magnification (100×), with valid results requiring detectable signals in >75% of tumor nuclei. The criteria were consistent with established guidelines (17).

### PD-L1 expression assessment and threshold selection

PD-L1 expression level for each patient was determined using the Dako 22C3 pharmDx system (Agilent Technologies Inc., Santa Clara, CA, USA) (18). Tissue samples were counterstained with hematoxylin according to the manufacturer’s instructions. PD-L1 positivity was defined as CPS≥1, where CPS was the number of PD-L1-positive cells (tumor cells, lymphocytes, and macrophages) divided by the total number of viable tumor cells multiplied by 100. The categorization of PD-L1 expression into CPS thresholds of 1, 5, and 10 was based on established clinical and molecular evidence, including data from the KEYNOTE-811 (10), Checkmate-649 (11), and KEYNOTE-859 (12) trials. Additionally, a further analysis was conducted to explore the potential significance of a CPS threshold of 20. Qualified pathologists performed cell counts and CPS categorization.

### Data collection and statistical analysis

All data for 478 GCs (435 with overall survival (OS) data and 342 with disease-free survival (DFS) data) used in this research were obtained from the public database cbiportal (https://www.cbioportal.org/datasets). We used the survfit function from the R package “survival” to analyze the differences in prognosis between the different groups of samples. We then assessed the significant prognostic difference between the groups using the log-rank test method on the Sangerbox platform (19) (http://www.sangerbox.com/tool, a free online platform for comprehensive data analysis).

An exploratory analysis using receiver operating characteristic (ROC) curves was performed to analyze the association between the number of copies of *HER2* by NGS and *HER2* status using FISH. The prevalence and distribution of genomic alterations were visualized using R package “maftools” (20). The R package “ggplot2” was used to draw box plots and stacked bar charts. Continuous variables were compared between the two groups using the Wilcoxon rank-sum test (Mann-Whitney U test), while categorical variables were analyzed with either the Chi-square test or Fisher’s exact test, depending on their distribution characteristics. For exploratory *post hoc* comparisons, multiple testing adjustments were performed using the Benjamini-Hochberg procedure to control the false discovery rate.

## Results

### Performance of detecting HER2 amplification based on NGS sequencing

In total, 390 patients diagnosed with GC were included in this study. The *HER2* amplification status of 254 GCs was determined using both FISH and NGS methods, and the results are shown in **Table 1**. FISH detected 33 GCs with *HER2* amplification, while NGS detected 32 GCs with amplification copies greater than 2.0, as detailed in **Supplementary Table S1**. FISH is considered the gold standard for *HER2* amplification in GC. To validate the performance of NGS in detecting *HER2* amplification, we defined the positive thresholds for NGS as 2.5, 3.0, 4.0, 5.0 and 6.0, and compared the results with FISH as the reference. In this study, the concordance rates were 99.6% in 2.5 copies, 98.4% in 3.0 copies, 92.1% in 4.0 copies, 91.3% in 5.0 copies, and 90.2% in 6.0 copies (**Table 1**). The sensitivity, specificity, positive predictive value (PPV), and negative predictive value (NPV) were calculated. The results are shown in **Supplementary Figure S1**. At 2.5 copies, NGS demonstrated high sensitivity (97.0%) and perfect specificity (100%), with a PPV of 99.5% and NPV of 100%. Increasing the threshold to 3.0 reduced sensitivity (87.9%) while maintaining 100% specificity, PPV (98.2%), and NPV (100%). Further threshold increments to 4.0, 5.0, and 6.0 led to progressive declines in sensitivity (39.4%, 33.3%, and 24.2%, respectively), though specificity remained 100%, with PPVs of 91.7–89.8% and sustained 100% NPV. Therefore, when the threshold was set to 2.5 copies, the results of NGS for detecting *HER2* amplification were highly consistent with those of FISH, serving as the threshold for subsequent positive *HER2* amplification detection by NGS.

**Table 1:** Comparison of consistency between different thresholds for NGS detection of *HER2* amplification-positive and FISH results.

Characteristics	ALL	*HER2* FISH		Concordance rate
		Negative	Positive	
	*N=254*	*N=221*	*N=33*	
*HER2*_NGS (copies≥2.5)				253/254 (99.6%)
Negative	222 (87.4%)	221 (100%)	1 (3.03%)	
Positive	32 (12.6%)	0 (0.00%)	32 (97.0%)	
*HER2*_NGS (copies≥3.0)				250/254 (98.4%)
Negative	225 (88.6%)	221 (100%)	4 (12.1%)	
Positive	29 (11.4%)	0 (0.00%)	29 (87.9%)	
*HER2*_NGS (copies≥4.0)				234/254(92.1%)
Negative	241 (94.9%)	221 (100%)	20 (60.6%)	
Positive	13 (5.12%)	0 (0.00%)	13 (39.4%)	
*HER2*_NGS (copies≥5.0)				232/254 (91.3%)
Negative	243 (95.7%)	221 (100%)	22 (66.7%)	
Positive	11 (4.33%)	0 (0.00%)	11 (33.6%)	
*HER2*_NGS (copies≥6.0)				229/254 (90.2%)
Negative	246 (96.9%)	221 (100%)	25 (75.8%)	
Positive	8 (3.15%)	0 (0.00%)	8 (24.2%)	

**Figure 1:**
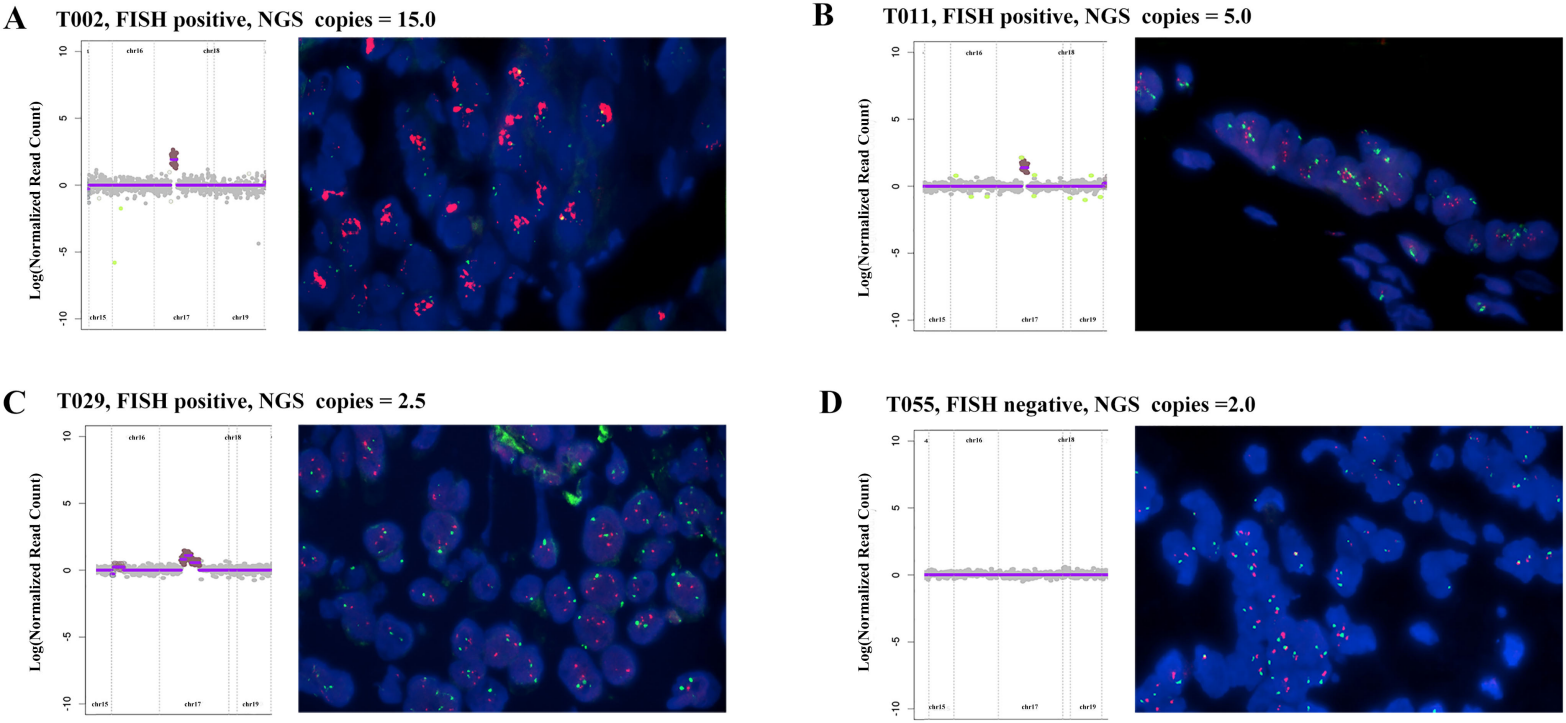
Comparison of *HER2* amplification between NGS detection and FISH results in four advanced gastric cancer patients: **(A)** FISH positive, NGS result: 15.0 copies. **(B)** FISH positive, NGS result: 5.0 copies; **(C)** FISH positive, NGS result: 2.5 copies; **(D)** FISH negative, NGS result: 2 copies; Red: HER2; Green: CEP17; Blue: DAPI.

**Figure 1** illustrates a comparison between *HER2* FISH test results and NGS test results, presenting four examples. In Case T002, the FISH test result was notably positive (red *HER2* signals exhibited in multiple clusters), with an NGS result of 15.0 copies (**Figure 1A**). Similarly, when the FISH result indicated clear positivity (red *HER2* signals appearing in multiple copies), the NGS result was 5 copies (**Figure 1B**, Case T011); in cases with weak positive FISH results, the NGS result was 2.5 copies (**Figure 1C**, Case T029). Conversely, in Case T055, the FISH test result was negative (red *HER2* signals without aggregation and dispersion), and the corresponding NGS result was recorded as two copies (**Figure 1D**). This suggests that in the majority of cases, NGS detection of *HER2* amplification aligns with FISH results, highlighting the reliability of NGS testing.

### Mutation overview and analysis with *HER2* amplification status

**Figure 2:**
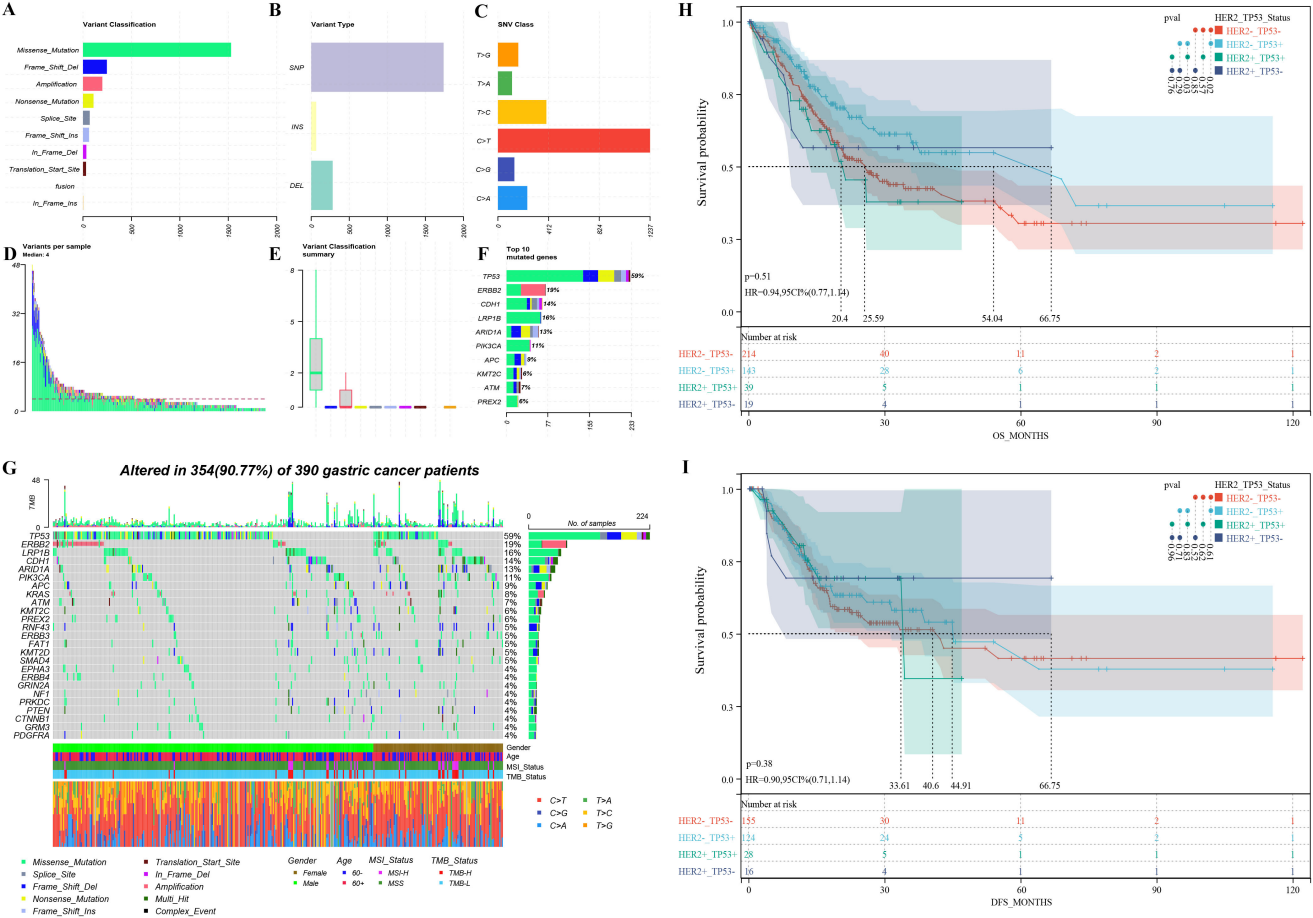
Mutation overview of 390 advanced gastric cancer patients **(A-G)** and survival differences among patients with varied *HER2* amplification and *TP53* mutation groups **(H-I)**: **(A)** The variant classification, **(B)** variant type, and **(C)** SNV class of mutated genes involved in GC tumors; **(D)** variants in each sample; **(E)** summary of variant classification; **(F)** Mutation types of the top 10 genes; **(G)** The waterfall diagram indicates the top 25 mutated genes and their variant types in GC tissues; overall survival **(H)** and disease-free survival **(I)** disparities among patients with distinct *HER2* amplification and *TP53* mutation groups. HER2+_TP53+: Patients with *HER2* amplification and *TP53* mutation. HER2+_TP53-: Patients with *HER2* amplification and without *TP53* mutation. HER2-_TP53+: Patients without *HER2* amplification and with *TP53* mutation. HER2-_TP53-: Patients without *HER2* amplification and *TP53* mutation.

All participants successfully completed targeted sequencing, which included all exons and partial introns of the 639 genes listed in **Supplementary Table S2**. Sequencing data from 390 samples were compiled, and detailed information can be accessed in **Supplementary Table S3**. Among the observed mutation types, missense mutations were the most common, followed by amplification and frameshift deletions (see **Figure 2A**). Single nucleotide polymorphisms (SNPs) constituted a larger proportion than insertions or deletions (**Figure 2B**). Notably, the C > T transition was the dominant single nucleotide variant (SNV) observed in GCs (**Figure 2C**). The number of altered bases in each sample and a summary of the variant classifications were counted, as shown in **Figure 2D** and **Figure 2E**, respectively. In GCs, the top 10 mutated genes were *TP53* (59%), *HER2* (19%), *LRP1B* (16%), *CDH1* (14%), *ARID1A* (13%), *PIK3CA* (11%), *APC* (9%), *ATM* (7%), *KMT2C* (6%), and *PREX2* (6%) (**Figure 2F**) which might play an important role in the biological processes of GC. According to the waterfall plot of the top 25 mutated genes, the mutation type is denoted by various colors with annotations; loss of function, gene amplification, and missense mutations were mostly observed (**Figure 2G**).

We further analyzed the association between *HER2* amplification status and clinical and genomic features. As shown in **Table 2**, the participants had an average age of 62.9 years (range: 28–88 years; median, 64 years) at the time of diagnosis, with no significant difference between the *HER2* amplification (copies ≥2.5) and non-amplification groups (p = 0.748). Similarly, there was no significant correlation between *HER2* amplification status and sex (p = 0.067). Given the highest mutation rate in *TP53*, we concurrently analyzed the association between *HER2* amplification status and *TP53*, revealing a significantly higher proportion of *TP53* mutations in the *HER2* amplification group than in the non-amplification group (P < 0.01). Similar results have been observed in other positive threshold values for *HER2* amplification detected by NGS (**Supplementary Table S4**). Furthermore, *HER2* amplification positivity and microsatellite instability-high (MSI-H) appeared to be mutually exclusive (p = 0.035), with no MSI-H cases identified among *HER2* amplification-positive (copies ≥2.5) patients. However, no significant differences in MSI values were observed between the *HER2* amplification and non-amplification groups (**Supplementary Figure S2B**). Nevertheless, *HER2* amplification status showed no significant correlation with tumor mutational burden (TMB) (**Table 2** and **Supplementary Figure S2A**), age (**Supplementary Figure S2C**), or tumor purity (**Supplementary Figure S2D**).

**Table 2:** Summary descriptive table grouped by *HER2* status detected by NGS.

Characteristics	ALL N=390	*HER2* NGS		OR	p.ratio	^a^p.overall
		Negative N=345	Positive N=45			
Gender						0.07
Female	110 (28.2%)	103 (29.9%)	7 (15.6%)	Ref.	Ref.	
Male	280 (71.8%)	242 (70.1%)	38 (84.4%)	2.27 [1.03;5.75]	0.040	
Age at diagnosis in years						0.75
60- (<60 years)	134 (34.4%)	120 (34.8%)	14 (31.1%)	Ref.	Ref.	
60+ (≥60 years)	256 (65.6%)	225 (65.2%)	31 (68.9%)	1.17 [0.61;2.37]	0.638	
*TP53*_Status						0.00
Mutated	228 (58.5%)	191 (55.4%)	37 (82.2%)	Ref.	Ref.	
Wild	162 (41.5%)	154 (44.6%)	8 (17.8%)	0.27 [0.11;0.58]	<0.001	
TMB_Status						0.10
TMB-H (≥10 muts/Mb)	38 (9.74%)	37 (10.7%)	1 (2.22%)	Ref.	Ref.	
TMB-L (<10 muts/Mb)	352 (90.3%)	308 (89.3%)	44 (97.8%)	4.64 [0.97;110]	0.056	
MSI_Status						0.04
MSI-H (≥40%)	30 (7.69%)	30 (8.70%)	0 (0.00%)	Ref.	Ref.	
NonMSI-H (<40%)	360 (92.3%)	315 (91.3%)	45 (100%)	[.;.]		

a P value are tested by Chi-square Test or Fisher Exact Test.

### Survival analysis based on *HER2* amplification and *TP53* mutation status

Based on the relationship between *HER2* amplification and *TP53* mutations, we further categorized patients based on their status and analyzed prognostic differences using public databases. GC patients with *HER2* amplification and *TP53* mutations had a significant shorter OS compared to patients with *HER2* amplification-negative and *TP53*-mutated tumors (20.4 months vs. 54.04 months, p = 0.03) (**Figure 2H**). However, there was no difference in median DFS (33.61 months vs. 44.91 months, p = 0.83) (**Figure 2I**). In addition, among *HER2* amplification-negative GC patients, those with *TP53* mutations had a significantly higher median OS compared to patients without *TP53* mutations (54.04 months vs. 25.59 months, p = 0.02) (**Figure 2H**). Similarly, there was no difference in median DFS (44.91 months vs. 40.6 months, p = 0.61) (**Figure 2I**).

### The association between clinical and genomic features and stratifications of PD-L1 expression

Considering the significance of PD-L1 expression in advanced GCs, we conducted separate analyses to explore the distinct clinical and genomic features associated with PD-L1 expression at CPS score thresholds of 1, 5, 10, and 20. Positivity for PD-L1 expression was determined using these thresholds. The detailed CPS scores are listed in **Supplementary Table S3**. Regardless of the positive threshold for PD-L1, *HER2* amplification was not significantly associated with PD-L1 expression (**Table 3**, **Supplementary Figure S3** and **Supplementary Table S5**). However, overall, *HER2* amplification positivity was associated with a lower proportion of PD-L1 positive expression compared to the negative group (33.9% (n= 117) vs. 20.0% (n= 9), p = 0.088), with the most noticeable difference observed at the threshold of 5 (**Supplementary Figure S3B**).

**Table 3:** Summary descriptive table grouped by CPS score of PD-L1 expression.

Characteristics	ALL *N=390*	PD-L1 Type(CPS≥1)		^a^p-value	PD-L1 Type (CPS≥5)		^a^p-value	PD-L1 Type (CPS≥10)		^a^p-value	PD-L1 Type (CPS≥20)		^a^p-value
		Negative	Positive		Negative	Positive		Negative	Positive		Negative	Positive	
		*N=200*	*N=190*		*N=264*	*N=126*		*N=325*	*N=65*		*N=350*	*N=40*	
Gender				1.000			0.82			0.12			0.02
Female	110 (28.2%)	56 (28.0%)	54 (28.4%)		73 (27.7%)	37 (29.4%)		86 (26.5%)	24 (36.9%)		92 (26.3%)	18 (45.0%)	
Male	280 (71.8%)	144 (72.0%)	136 (71.6%)		191 (72.3%)	89 (70.6%)		239 (73.5%)	41 (63.1%)		258 (73.7%)	22 (55.0%)	
Age at diagnosis in years				0.06			0.08			0.60			0.54
60- (<60 years)	134 (34.4%)	78 (39.0%)	56 (29.5%)		99 (37.5%)	35 (27.8%)		114 (35.1%)	20 (30.8%)		118 (33.7%)	16 (40.0%)	
60+ (≥60 years)	256 (65.6%)	122 (61.0%)	134 (70.5%)		165 (62.5%)	91 (72.2%)		211 (64.9%)	45 (69.2%)		232 (66.3%)	24 (60.0%)	
*TP53*_Status:				0.36			0.26			0.04			0.10
Mutated	228 (58.5%)	112 (56.0%)	116 (61.1%)		160 (60.6%)	68 (54.0%)		198 (60.9%)	30 (46.2%)		210 (60.0%)	18 (45.0%)	
Wild	162 (41.5%)	88 (44.0%)	74 (38.9%)		104 (39.4%)	58 (46.0%)		127 (39.1%)	35 (53.8%)		140 (40.0%)	22 (55.0%)	
TMB_Status				0.17			0.04			0.02			0.04
TMB-H (≥10 muts/Mb)	38 (9.74%)	15 (7.50%)	23 (12.1%)		20 (7.58%)	18 (14.3%)		26 (8.00%)	12 (18.5%)		30 (8.57%)	8 (20.0%)	
TMB-L (<10 muts/Mb)	352 (90.3%)	185 (92.5%)	167 (87.9%)		244 (92.4%)	108 (85.7%)		299 (92.0%)	53 (81.5%)		320 (91.4%)	32 (80.0%)	
MSI_Status				0.06			0.01			0.02			0.02
MSI-H (≥40%)	30 (7.69%)	10 (5.00%)	20 (10.5%)		14 (5.30%)	16 (12.7%)		20 (6.15%)	10 (15.4%)		23 (6.57%)	7 (17.5%)	
NonMSI-H (<40%)	360 (92.3%)	190 (95.0%)	170 (89.5%)		250 (94.7%)	110 (87.3%)		305 (93.8%)	55 (84.6%)		327 (93.4%)	33 (82.5%)	
*HER2*_NGS				0.44			0.06			0.20			0.29
Negative	345 (88.5%)	174 (87.0%)	171 (90.0%)		228 (86.4%)	117 (92.9%)		284 (87.4%)	61 (93.8%)		307 (87.7%)	38 (95.0%)	
Positive	45 (11.5%)	26 (13.0%)	19 (10.0%)		36 (13.6%)	9 (7.14%)		41 (12.6%)	4 (6.15%)		43 (12.3%)	2 (5.00%)	

a p-value are tested by Chi-square Test or Fisher Exact Test.

As shown in **Table 3**, PD-L1 positivity was often associated with a higher proportion of TMB-high (TMB-H) status, with significant differences observed at CPS thresholds of 5, 10 and 20 (p <0.05). However, regardless of the CPS threshold, there were no significant differences in TMB values among the different PD-L1 expression levels (**Supplementary Figure S4A**). Similarly, PD-L1 positivity was often associated with a higher proportion of MSI-high (MSI-H) (**Table 3**), with significant differences observed at CPS thresholds of 5, 10, and 20 (p <0.05). There were no significant differences in MSI values among the different PD-L1 expression levels (**Supplementary Figure S4B**). Although the proportion of age stratification did not differ among the different PD-L1 stratifications (**Table 3**), there were significant differences in age among the different levels of PD-L1 expression when the threshold was set at 1 or 5 (64.4 vs. 61.4, P < 0.01; 64.8 vs. 61.9, P < 0.01) (**Supplementary Figure S4C**). Regardless of how PD-L1 was stratified, the level of PD-L1 expression was positively correlated with tumor purity, indicating that PD-L1-positive expression was associated with higher tumor purity (**Supplementary Figure S4D**). Unlike the relationship between *HER2* amplification and *TP53* mutation status, the relationship between PD-L1 expression and *TP53* mutation status depends on the CPS threshold. When the threshold was set at 1 or 5, PD-L1-positive expression was often associated with a higher proportion of *TP53* mutations, whereas this relationship was reversed when the threshold was set at 10 or 20 (**Table 3**). Additionally, there was a higher proportion of male patients with PD-L1-positive expression (**Table 3**), with a significant difference observed at a threshold of 20 (55.0% vs. 45.0%, p = 0.021).

### The analysis of gene mutation in different stratifications of PD-L1 expression

**Figure 3:**
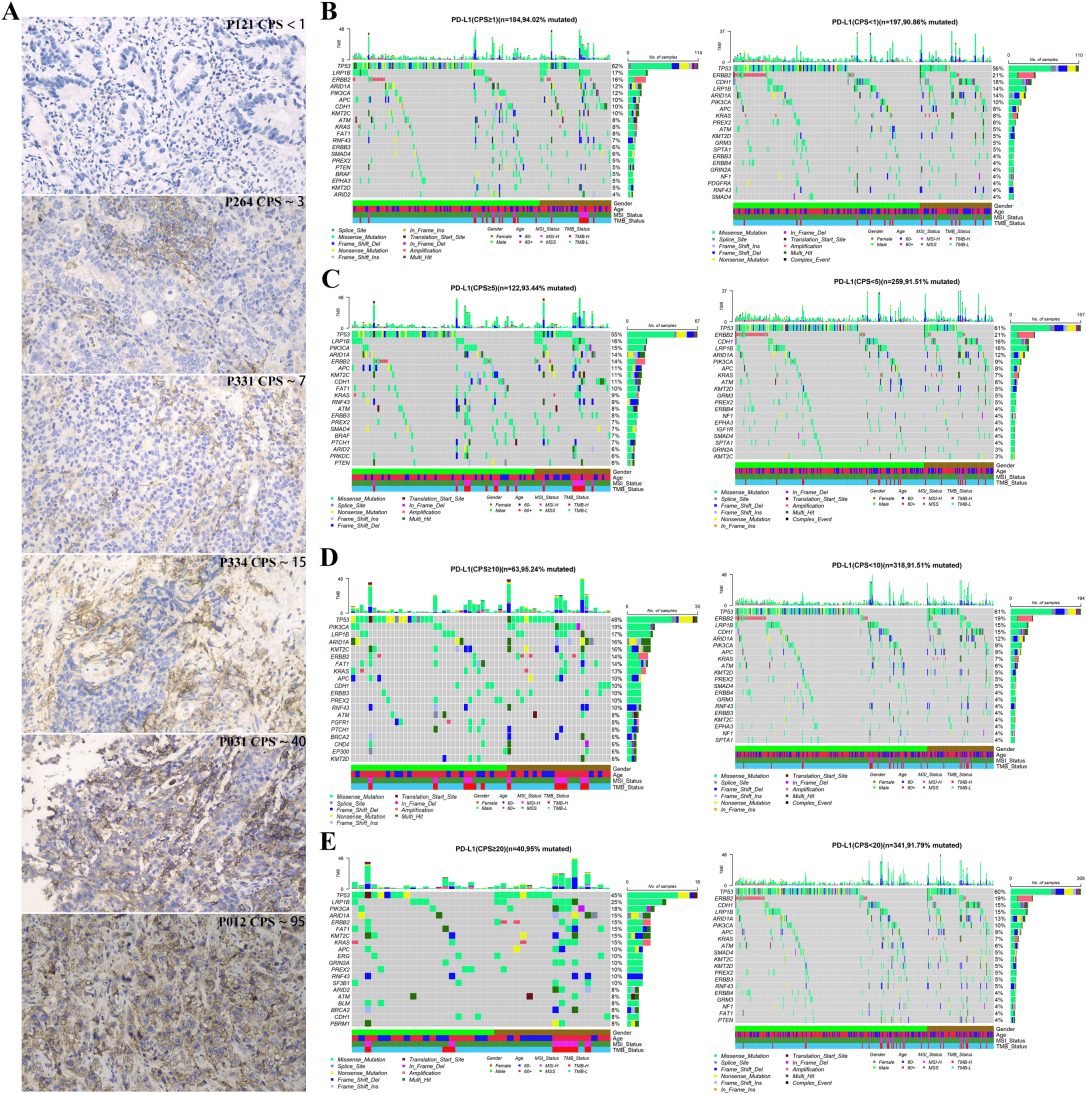
Gene mutation landscape of advanced gastric cancer patients with different PD-L1 expression groups at CPS score thresholds: **(A)** Immunohistochemical image of gastric cancer patients with different expression of PD-L, 200X; The top 20 mutated genes in different PD-L1 expression groups with CPS score thresholds of 1 **(B)**, 5 **(C)**, 10 **(D)**, and 20 **(E)**.

**Figure 3A** illustrates the PD-L1 staining results of six representative gastric cancer patients corresponding to different expression levels. To understand the reasons for the variations in clinical indicators among distinct PD-L1 subgroups, we analyzed the genetic mutation characteristics within these subgroups. As depicted in **Figures 3B–E**, there were differences in the types and mutation rates of 20 mutated genes among patients in different PD-L1 subgroups.

**Figure 4:**
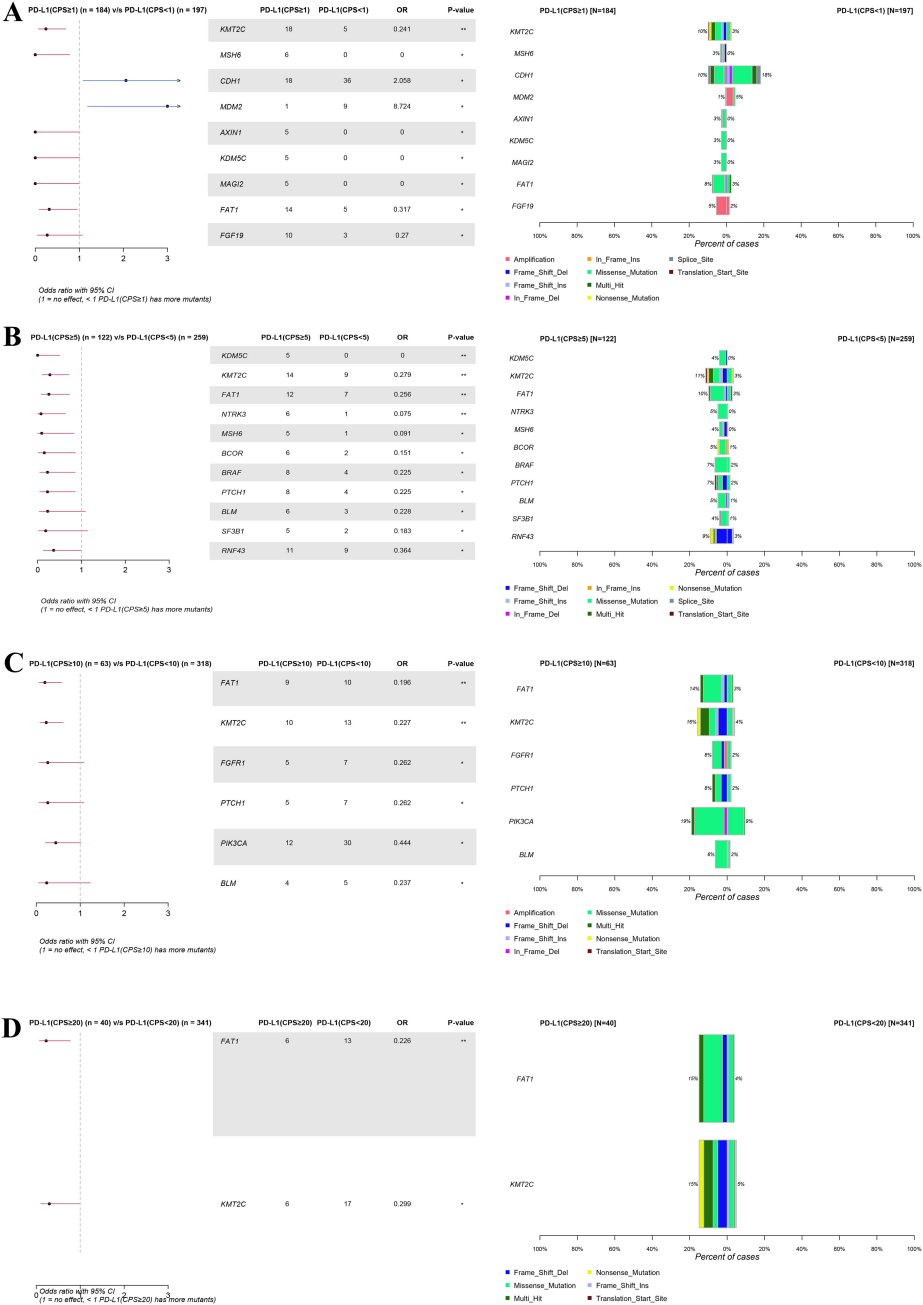
Forest plot and co-bar plot comparing differentially mutated genes between gastric cancer patients with different expression groups of PD-L1 at CPS score thresholds of 1 **(A)**, 5 **(B)**, 10 **(C)**, and 20 **(D)**. “*” and “**” indicate P < 0.05 and P < 0.01, Fisher’s exact test.

Notably, when the CPS threshold was set to 1 (**Figure 4A**), significant differences (P < 0.05) were observed in the gene mutations, including *KMT2C* (10% vs. 3%), CDK6(0% vs. 4%), *MSH6* (3% vs. 0%), *CDH1* (10% vs. 18%), *MDM2* (1% vs. 5%), *AXIN1* (3% vs. 0%), *KDM5C* (3% vs. 0%), *MAGI2* (3% vs. 0%), and *FAT1* (8% vs. 3%). When the CPS threshold was set at 5 (**Figure 4B**), significant differences were observed in gene mutations, including *KDM5C* (4% vs. 0%), *KMT2C* (11% vs. 3%), *FAT1* (10% vs. 3%), *NTRK3* (5% vs. 0%), *MSH6* (4% vs. 0%), *BCOR* (5% vs. 1%), *BRAF* (7% vs. 2%), *PTCH1* (7% vs. 2%), *BLM* (5% vs. 1%), *SF3B1* (4% vs. 1%), and *RNF43* (9% vs. 3%). Similarly, when the CPS threshold was set at 10 (**Figure 4C**), significant differences were observed in the expression of genes including *FAT1* (14% vs. 3%), *KMT2C* (16% vs. 4%), *FGFR1* (8% vs. 2%), *PTCH1* (8% vs. 2%), *PIK3CA* (19% vs. 9%), *BLM* (6% vs. 2%), and *EP300* (8% vs. 3%). Finally, when the CPS threshold was set at 20 (**Figure 4D**), significant differences were observed in the expression of genes including *FAT1* (15% vs. 4%) and *KMT2C* (15% vs. 5%).

### Tumor signaling pathway analysis in different stratifications of PD-L1 expression

**Figure 5:**
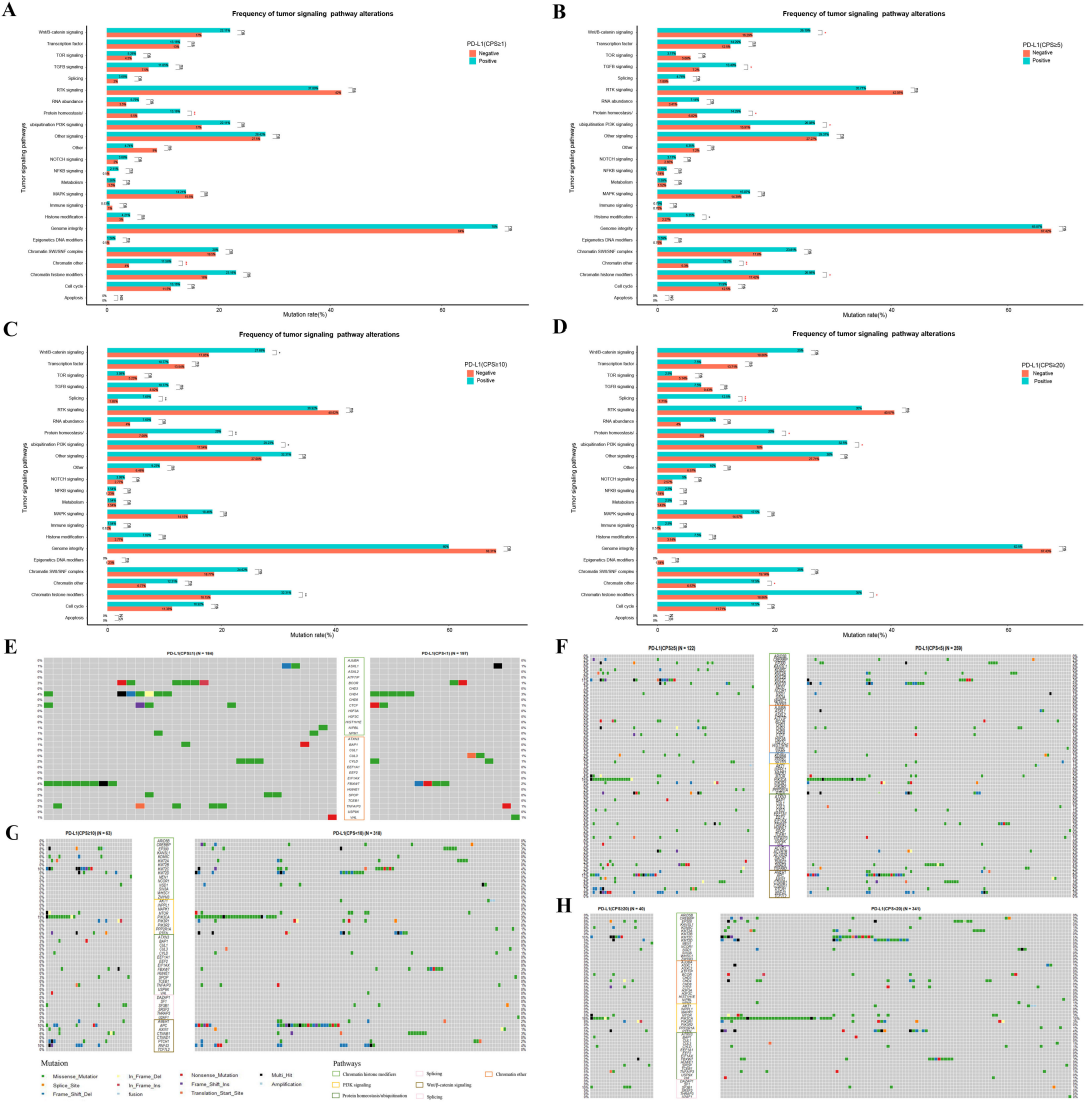
Differential mutations analysis in the enrichment of tumor signaling pathway-related genes between GC patients with different PD-L1 expression groups of PD-L1 at CPS score thresholds of 1 **(A, E)**, 5 **(B, F)**, 10 **(C, G)**, and 20 **(D, H)**. “ns”, “*”, “**” and “***” indicate P > 0.05, P < 0.05, P < 0.01 and P < 0.001, Wilcox test; Exploratory analyses with Benjamini-Hochberg-adjusted p-values in **Supplementary Table S6-9**; see Methods for analysis definitions.

To further investigate the potential impact of different PD-L1 expression levels on tumor signaling pathways in patients with GC, we conducted a mutation enrichment analysis of tumor signaling pathways. The pathways and related genes referenced in **Supplementary Table S3** in a previous study (21) were utilized for this analysis. As shown in **Figure 5** and **Supplementary Tables S6-S9**, different levels of PD-L1 expression demonstrated variations in mutations across various pathways. When the CPS threshold was set to 1, positive PD-L1 expression was associated with a higher mutation rate in the chromatin other, protein homeostasis, and ubiquitination pathways (11.58% vs. 4.00%, P < 0.01; 13.16% vs. 5.50%, P < 0.01) (**Figure 5A**). However, the mutation rate difference in the related genes was not pronounced (**Figure 5E**). When the CPS threshold was set to 5, positive PD-L1 expression was linked to an increased mutation rate in the chromatin histone modifiers, chromatin modification, histone modification, PI3K signaling, protein homeostasis/ubiquitination, TGFβ signaling, and Wnt/β-catenin signaling pathways (26.98% vs. 14.72%, P < 0.05; 12.70% vs. 5.30%, P < 0.01; 6.35% vs. 2.27%, P < 0.05; 26.98% vs. 15.91%, P < 0.05; 14.29% vs. 6.82%, P < 0.05; 13.49% vs. 7.20%, P < 0.01; 26.19% vs. 16.29%, P < 0.05) (**Figure 5B**). Notably, the mutation rate differences in the *KMT2C, PIK3CA, PTCH1 and RNF43* genes were pronounced (11% vs. 3%, 15% vs. 9%, 7% vs. 2%, and 9% vs. 3%) (**Figure 5F**). When the CPS threshold was set to 10, positive PD-L1 expression was associated with a higher mutation rate in the chromatin histone modifiers, PI3K signaling, protein homeostasis/ubiquitination, splicing, and Wnt/β-catenin signaling pathways (32.31% vs. 18.15%, P < 0.01; 29.23% vs. 17.54%, P < 0.05; 20.00% vs. 7.08%, P < 0.01; 7.69% vs. 1.85%, P < 0.01; 27.69% vs. 17.85%, P < 0.05) (**Figure 5C**). *EP300*, *KDM5C, KMT2C, PIK3CA, PTCH1 and RNF43* showed significant differences in mutation rates (8% vs. 3%, 6% vs. 0%, 16% vs. 4%, 19% vs. 9%, 8% vs. 2%, 10% vs. 4%) (**Figure 5G**). When the CPS threshold was set to 20, positive PD-L1 expression was associated with a higher mutation rate in chromatin histone modifiers, chromatin others, PI3K signaling, protein homeostasis/ubiquitination, and splicing pathways (35.00% vs. 18.86%, P < 0.05; 17.50% vs. 6.57%, P < 0.05; 32.50% vs. 18.00%, P < 0.05; 20.00% vs. 8.00%, P < 0.05; 12.50% vs. 1.71%, P < 0.001) (**Figure 5D**). It is worth mentioning that the mutation rates of *EP300*, *KDM5C*, *KMT2C*, *PIK3CA*, *PIK3R1* and *SF3B1* were significantly different (8% vs. 3%, 8% vs. 1%, 15% vs. 5%, 18% vs. 10%; 8% vs. 3%, 10% vs. 1%) (**Figure 5H**).

## Discussion

*HER2* and PD-L1 are important targets in GC. FISH testing for *HER2* amplification is considered the gold standard (22), but it can be complex to perform and is subject to some degree of interpretation subjectivity. The role of NGS in cancer gene testing is becoming increasingly evident. In this study, we examined the concordance between NGS detection of *HER2* amplification and FISH results in GCs for the first time. When the positive threshold for NGS testing was set at 2.5, it showed the highest concordance with the FISH results (sensitivity, 97.0%; specificity, 100%; PPV, 99.5%; NPV, 100%). This result indicates the reliability of NGS detection of *HER2* amplification, which is consistent with previous research results in other cancers (23, 24), suggesting that it can replace FISH testing to some extent in GCs.

Furthermore, we conducted a retrospective analysis of the gene mutation information in 390 GC cases. We found that patients with *HER2* amplification positive status had a higher proportion of *TP53* mutations. Through analysis of public databases, we observed that among *TP53*-positive GCs, patients with *HER2* amplification positive status had a significantly shorter median OS time than patients with *HER2* amplification negative status. This suggests that GC patients with *HER2*-positive and *TP53*-mutated may require different treatment approaches compared to other patients, such as combined anti-HER2 antibody (3, 7) and p53 activators, such as APR-246 (25, 26). Additionally, our results reveal a striking mutual exclusivity between *HER2* amplification and MSI-H status (0/45 in HER2+ vs 30/345 in HER2- cases, p=0.035). This finding is consistent with both the exceptionally low co-occurrence rate (0.7%) reported in KEYNOTE-811 (4) and the fundamentally distinct molecular profiles of these tumor subtypes: while *HER2-*amplified tumors typically exhibit chromosomal instability with low mutational burden, MSI-H tumors display mismatch repair deficiency-driven hypermutability without recurrent amplifications (27).

Although *HER2* and PD-L1 are both important targets, according to our data analysis, they appeared to have an antagonistic relationship, especially when the CPS threshold was set at 5, when this difference was most pronounced (p=0.088). This finding suggests that *HER2* amplification and PD-L1 expression are independent indicators of gastric cancer. Specifically, *HER2*-positive tumors tended to have low PD-L1 expression, whereas *HER2*-negative tumors tended to have high PD-L1 expression. These findings align with previous research (28). It is puzzling that, in one study, the expression of PD-L1 and HER2 in gastric cancer patients was positively correlated (17). Pathological analysis of the spatial distribution of PD-L1 and HER2-positive tumor regions reveals little overlap, further suggesting that these two factors may promote tumor progression through different mechanisms (29). In gastric cancer, approximately 10% of patients exhibit *HER2* amplification, and around 40% show PD-L1 positivity, yet only 4.5% are positive for both markers (30). Importantly, the simultaneous overexpression of HER2 and PD-L1 is an independent prognostic factor associated with poorer survival outcomes (17). The combination of anti-HER2 therapy, such as trastuzumab, and anti-PD-L1 therapy, such as pembrolizumab, has been shown to significantly improve survival in patients who are PD-L1 positive and have HER2 overexpression (4, 31). However, existing studies suggest that activation of the PD-1/PD-L1 signaling axis can contribute to both inherent and acquired resistance to HER2-targeted therapies (e.g., trastuzumab) (32). HER2-positive tumors can inhibit T-cell activity by upregulating PD-L1 expression, creating an immunosuppressive tumor microenvironment (33). Some research indicates that the HER2 signaling pathway may indirectly regulate PD-L1 expression, possibly through activation of the PI3K/AKT/mTOR pathway or by promoting the secretion of immunosuppressive cytokines in the tumor microenvironment (34). This discrepancy between result of our research and previous studies requires further investigation.

Our findings build upon previous reports demonstrating threshold-dependent responses to PD-L1 inhibitors (10–14), by revealing distinct biological and clinical implications at different PD-L1 expression levels. The most clinically relevant associations emerged at CPS thresholds of 5, 10, and 20, where PD-L1 positivity correlated with TMB-H and MSI-H status - biomarkers known to influence immunotherapy response. Interestingly, these associations were absent at CPS=1, suggesting this lower threshold may lack sufficient discriminatory power for clinical decision-making. While we observed no statistically significant differences in TMB and MSI values between PD-L1 positive and negative groups, the consistent trend of elevated biomarker levels in PD-L1 positive patients warrants further investigation. The prognostic implications of these findings are particularly intriguing, as high PD-L1 expression coupled with TMB-H status (17) or MSI-H status (35) may represent distinct biological subsets with different clinical outcomes. These observations highlight the complex interplay between PD-L1 expression and other immunotherapy biomarkers in gastric cancer, emphasizing the need for comprehensive biomarker profiling rather than reliance on PD-L1 expression alone for treatment stratification.

Our molecular profiling revealed distinct genomic and pathway alterations associated with different PD-L1 CPS thresholds, offering insights for immunotherapy stratification in gastric cancer. At CPS=1, we identified mutations in *MAGI2*, *AXIN1*, *MDM2*, *CDH1*, and *CDK6*, while CPS=5 was associated with *RNF43*, *SF3B1*, *BRAF*, *BCOR*, MSH6, *NTRK3*, and *KDM5C* alterations. The CPS=10 threshold showed *EP300*, *PIK3CA*, and *FGFR1* mutations, whereas no specific gene associations emerged at CPS=20, suggesting diminished molecular discrimination at higher thresholds. Importantly, pathway analysis at CPS=5 revealed enrichment in Wnt/β-catenin, PI3K, and TGF-β signaling - pathways with established immunotherapy implications. TGF-β reshapes the tumor microenvironment by expanding Treg cells, suppressing CD8+ T and NK cell activity, and modulating MDSCs and macrophages, thereby promoting immune evasion and dampening anti-tumor immunity (36). Targeting TGF-β signaling enhances the efficacy of anti-PD-1/PD-L1 therapy, making it a key focus in cancer immunotherapy. Similarly, in the PI3K pathway, *PIK3CA* mutations and *PTEN* loss can lead to PD-L1 upregulation through AKT/mTOR-dependent transcriptional activation (37–39). Nevertheless, further *in vivo* and *in vitro* mechanistic studies are required to confirm these associations. Notably, β-catenin was identified as a transcription factor for PD-L1. Aberrant activation of the Wnt/β-catenin signaling pathway can undermine cancer immunosurveillance. Suppression of Wnt/β-catenin signaling by ISG12a downregulates PD-L1 expression, thereby enhancing the susceptibility of cancer cells to NK cell-mediated cytotoxicity (40). These findings collectively suggest that intermediate PD-L1 thresholds (particularly CPS=5) may optimally balance molecular discriminative power and clinical relevance for immunotherapy patient selection, though functional validation of these pathway interactions remains warranted.

Notably, our study had certain limitations. First, as a retrospective analysis, our single-center cohort lacks comprehensive clinical and prognostic variables (such as treatment history, comorbidities, etc.). Prognostic analysis was conducted using the TCGA-STAD database, which includes diverse populations, but our data only represents an Asian cohort. This discrepancy may introduce potential bias. Second, the absence of trastuzumab treatment records in the TCGA cohorts is a key limitation, as it highlights the poorer survival observed in HER2+ patients, particularly in the pre-trastuzumab era. This likely reflects HER2’s intrinsic prognostic role in untreated gastric cancer, as *HER2* amplification typically predicts improved survival with HER2-targeted therapies. Additionally, factors like tumor stage, age, and performance status may confound survival analysis. While our dataset lacks sufficient clinical information for multivariate adjustment, we performed a subgroup analysis on the *TP53* mutation group to reduce heterogeneity. Future studies integrating treatment-response data, especially for anti-HER2 and immunotherapy combinations, and incorporating detailed clinical annotations, are essential to validate these findings. Furthermore, our analysis of PD-L1 expression differences focused solely on molecular characteristics (gene mutations), leaving mechanistic insights for future investigation. While no CPS threshold of 20 has been established for gastric cancer, understanding distinct PD-L1 CPS expression patterns across malignancies, as highlighted in trials like CM649 and KEYNOTE-859, is crucial. Additionally, the TAP score introduced in the Rationale trial provides a valuable framework for evaluating PD-L1 expression and therapeutic responses, which could enhance future analyses. Finally, the single-center, retrospective design and predominantly Asian cohort limit the generalizability of our findings. Genetic factors, lesion location, dietary habits, and regional diagnostic practices may influence the HER2-PD-L1 association. Multicenter, prospective studies involving diverse ethnic populations are needed to validate and extend our conclusions.

## Conclusions

In summary, our research demonstrates the feasibility of NGS for *HER2* amplification in advanced gastric cancer. We identified the prognostic value of *HER2* amplification with *TP53* mutation, which provides valuable guidance for the precise treatment of such patients. Additionally, we observed differences in the clinical and molecular characteristics among different PD-L1 expression levels, which can serve as a reference for subsequent clinical studies focusing on gastric cancer patients with varying levels of PD-L1 expression.

## Data Availability

The original contributions presented in the study are included in the article and/or **Supplementary Material**; further inquiries can be directed to the corresponding authors.
