# Supplementary material for: Decoding the molecular landscape: HER2 and PD-L1 in advanced gastric cancer
Source: Front Immunol. 2025 May 30;16:1567308. doi: 10.3389/fimmu.2025.1567308 (PMC12163011; doi:10.3389/fimmu.2025.1567308)
Supplement: Supplementary file 1 [file DataSheet1.docx]

**
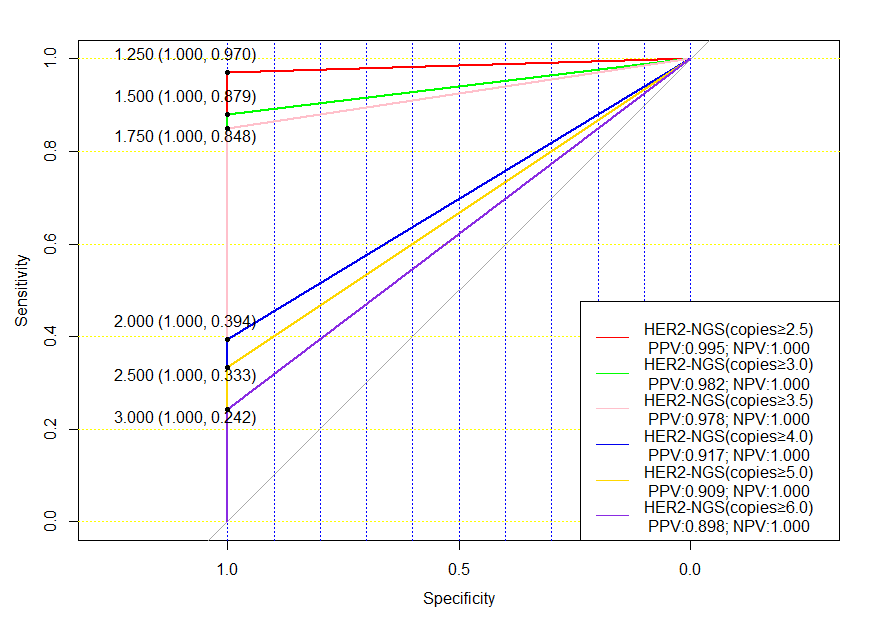
**

**Figure S1 ROC curves for the performance of NGS in detecting HER2 amplification at positive thresholds of 2.5, 3.0, 3.5, 4.0, 5.0 and 6.0**

PPV: positive predictive value; NPV: negative predictive value


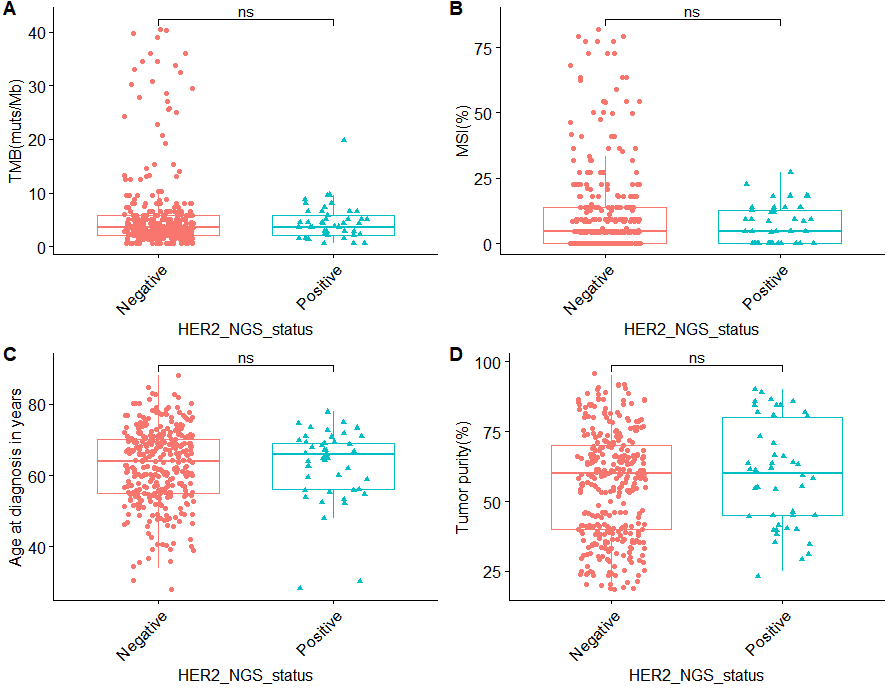


**Figure S2. Differential analysis of TMB (A), MSI value (B), age (C), and tumor purity (D) in between *HER2* amplification positive and negative groups.**

“ns” indicates P > 0.05, Wilcox test


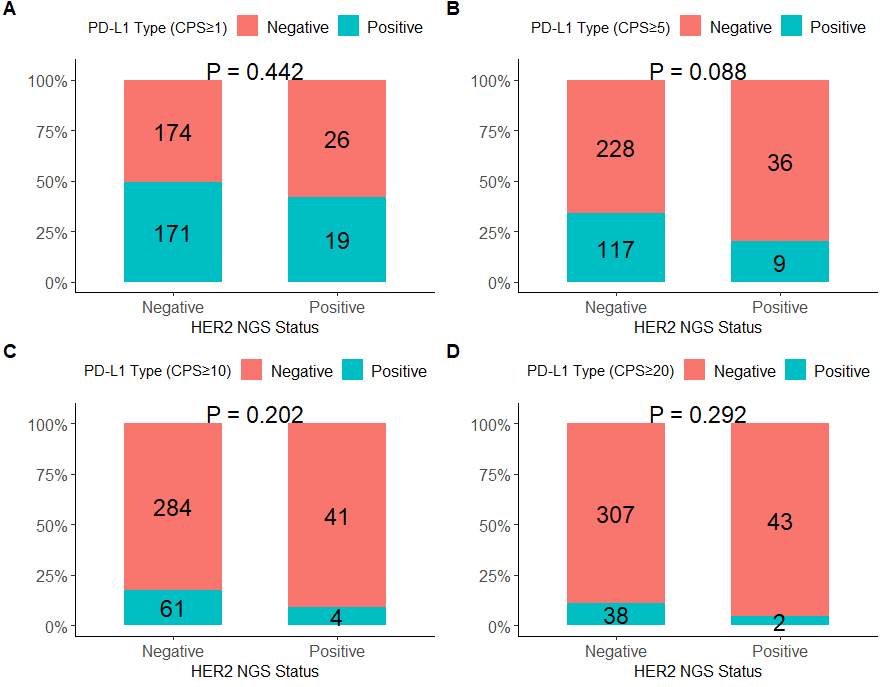


**Figure S3 Analysis of the relationship between PD-L1 expression and *HER2* amplification at CPS score thresholds of 1 (A), 5 (B), 10 (C), and 20 (D)**

P values are tested by the chi-square test


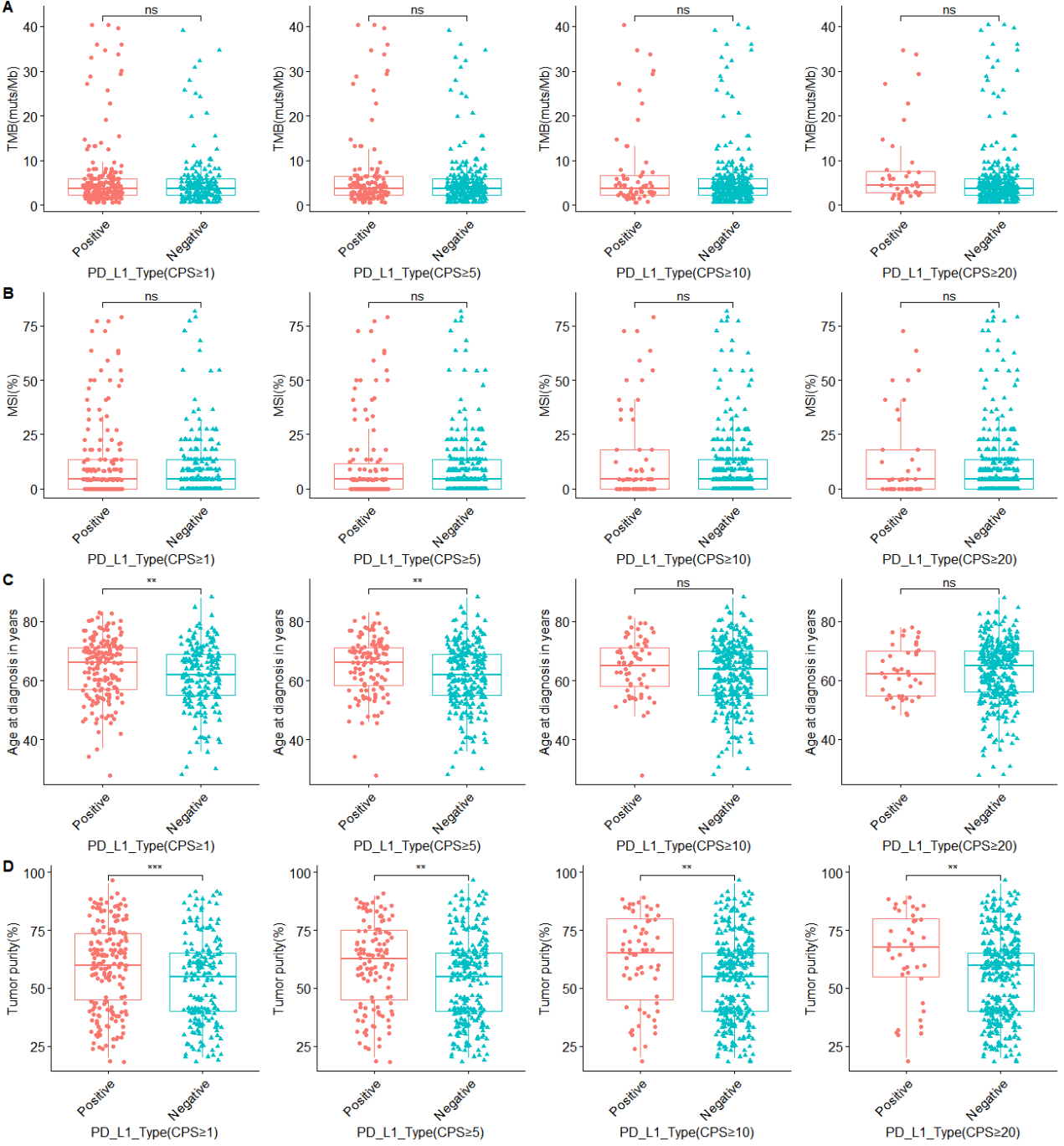


**Figure S4. The difference analysis of TMB (A), MSI (B), age (C), and tumor purity (D) in different PD-L1 expression groups with CPS score thresholds of 1, 5, 10, and 20**

“ns”, “**” and “***” indicate P > 0.05, P < 0.01 and P < 0.001, Wilcox test

**Table S1 Detailed Information on the NGS detection results of *HER2* amplification-positive gastric cancer samples and their FISH Results.**

| **Patient ID** | ***HER2* copies (NGS)** | ***HER2* status**  **(FISH)** |
| --- | --- | --- |
| T001 | 34.5 | Positive |
| T002 | 15 | Positive |
| T003 | 12.5 | Positive |
| T004 | 12.5 | Positive |
| T005 | 10 | Positive |
| T006 | 9.5 | Positive |
| T007 | 6 | Positive |
| T008 | 6 | Positive |
| T009 | 5.5 | Positive |
| T010 | 5.5 | Positive |
| T011 | 5 | Positive |
| T012 | 4 | Positive |
| T013 | 4 | Positive |
| T014 | 3.5 | Positive |
| T015 | 3.5 | Positive |
| T016 | 3.5 | Positive |
| T017 | 3.5 | Positive |
| T018 | 3.5 | Positive |
| T019 | 3.5 | Positive |
| T020 | 3.5 | Positive |
| T021 | 3.5 | Positive |
| T022 | 3.5 | Positive |
| T023 | 3.5 | Positive |
| T024 | 3.5 | Positive |
| T025 | 3.5 | Positive |
| T026 | 3.5 | Positive |
| T027 | 3.5 | Positive |
| T028 | 3.5 | Positive |
| T029 | 3 | Positive |
| T030 | 2.5 | Positive |
| T031 | 2.5 | Positive |
| T032 | 2.5 | Positive |

**Table S2.** The gene list of AllNGS-Panel 639^TM^

| **Gene name** | | | | | | | | | | | | | | |
| --- | --- | --- | --- | --- | --- | --- | --- | --- | --- | --- | --- | --- | --- | --- |
| *ABCA6* | *ABCB1* | *[ABCC11](https://www.snpedia.com/index.php/ABCC11)* | *ABCC2* | *ABCC4* | *ABCF1* | *ABCG2* | *ABL1* | *ABL2* | *ACTR3B* | *ACVR1B* | *ADAMTS10* | *ADNP* | *AGAP9* | *AHNAK* |
| *AKAP7* | *AKR1C2* | *AKT1* | *AKT2* | *AKT3* | *ALK* | *AMER1* | *ANK2* | *ANKRD36* | *ANO10* | *APC* | *AR* | *ARAF* | *ARFRP1* | *ARHGAP5* |
| *ARID1A* | *ARID1B* | *ARID2* | *ART5* | *ASPM* | *ASXL1* | *ATM* | *ATR* | *ATRX* | *AURKA* | *AURKB* | *AXIN1* | *AXL* | *BAP1* | *BARD1* |
| *BAX* | *BCL2* | *BCL2L1* | *BCL2L11* | *BCL2L2* | *BCL6* | *BCL6B* | *BCOR* | *BCORL1* | *BCR* | *BEND5* | *BLM* | *BMPR2* | *BRAF* | *BRCA1* |
| *BRCA2* | *BRD3* | *BRD4* | *BRIP1* | *BTG1* | *BTK* | *C11orf30* | *C1orf144* | *C21orf58* | *C22orf31* | *C8orf34* | *CARD11* | *CASP5* | *CBFB* | *CBL* |
| *CBR3* | *CBWD6* | *CCDC144NL* | *CCKBR* | *CCND1* | *CCND2* | *CCND3* | *CCNE1* | *CCR5* | *CD274* | *CD3G* | *CD79A* | *CD79B* | *CDA* | *CDC27* |
| *CDC42EP1* | *CDC7* | *CDC73* | *CDCP2* | *CDH1* | *CDH5* | *CDK12* | *CDK4* | *CDK6* | *CDK8* | *CDKN1A* | *CDKN1B* | *CDKN2A* | *CDKN2B* | *CDKN2C* |
| *CEBPA* | *CENPH* | *CEP162* | *CEP164* | *CHD2* | *CHD4* | *CHEK1* | *CHEK2* | *CIC* | *CLASP1* | *CLDN16* | *CLIP1* | *CLOCK* | *CNDP1* | *COBLL1* |
| *COL5A3* | *CREBBP* | *CRKL* | *CRLF2* | *CROCC* | *CSF1R* | *CSMD3* | *CTCF* | *CTNNA1* | *CTNNB1* | *CUL3* | *CUZD1* | *CYB5R4* | *CYLD* | *CYP19A1* |
| *CYP1B1* | *CYP21A2* | *CYP2C19* | *CYP2C8* | *CYP2D6* | *CYP3A4* | *CYP3A5* | *CYP4B1* | *DAXX* | *DCP1B* | *DDHD1* | *DDR2* | *DDX11* | *DDX23* | *DEFB126* |
| *DHFR* | *DHX8* | *DICER1* | *DIEXF* | *DLEC1* | *DNMT3A* | *DOT1L* | *DPYD* | *DYNC2H1* | *EBPL* | *EGFR* | *EHBP1* | *ELFN1* | *EP300* | *EPCAM* |
| *EPHA3* | *EPHA5* | *EPHA7* | *EPHB1* | *ERBB2* | *ERBB3* | *ERBB4* | *ERCC1* | *ERCC2* | *ERG* | *ERRFI1* | *ESR1* | *ESR2* | *ESRRA* | *EZH2* |
| *F2RL2* | *FAM174B* | *FAM186A* | *FAM46C* | *FAM71E2* | *FANCA* | *FANCC* | *FANCD2* | *FANCE* | *FANCF* | *FANCG* | *FANCL* | *FAS* | *FAT1* | *FBXW7* |
| *FCAMR* | *FCGBP* | *FCGR3A* | *FCRLA* | *FGF10* | *FGF14* | *FGF19* | *FGF23* | *FGF3* | *FGF4* | *FGF6* | *FGFR1* | *FGFR2* | *FGFR3* | *FGFR4* |
| *FH* | *FKBP9* | *FLCN* | *FLT1* | *FLT3* | *FLT4* | *FMN2* | *FOPNL* | *FOXL2* | *FOXP1* | *FRS2* | *FUBP1* | *GABRA6* | *GATA1* | *GATA2* |
| *GATA3* | *GATA4* | *GATA6* | *GCNT2* | *GGH* | *GGT1* | *GID4* | *GLI1* | *GLTSCR1* | *GNA11* | *GNA13* | *GNAQ* | *GNAS* | *GNLY* | *GOLGA6L4* |
| *GOLGA6L6* | *GOT1L1* | *GPR124* | *GRIK2* | *GRIN2A* | *GRM3* | *GSK3B* | *GSTM5* | *[GSTO1](https://www.snpedia.com/index.php/Special:FormEdit/Gene/GSTO1)* | *GSTP1* | *H3F3A* | *HAVCR1* | *HGF* | *HIF1A* | *HIST1H3B* |
| *HLA* | *HMGXB4* | *HNF1A* | *HNRNPL* | *HRAS* | *HSD3B1* | *HSP90AA1* | *HSPA8* | *[HTR1E](https://www.snpedia.com/index.php/HTR1E)* | *IDH1* | *IDH2* | *IFI27* | *IFITM3* | *IGF1R* | *IGF2* |
| *IKBKE* | *IKZF1* | *IL7R* | *INHBA* | *INPP4B* | *IRF2* | *IRF4* | *IRS2* | *ISX* | *ITPA* | *JAK1* | *JAK2* | *JAK3* | *JPH4* | *JUN* |
| *KANK3* | *KAT6A* | *KAT6B* | *KCNB2* | *KCNJ5* | *KDM5A* | *KDM5C* | *KDM6A* | *KDR* | *KEAP1* | *KEL* | *KIAA0355* | *KIAA1024* | *KIAA1211* | *KIF25* |
| *KIF6* | *KIT* | *KLF4* | *KLHL6* | *KMT2A* | *KMT2C* | *KMT2D* | *KPNA2* | *KRAS* | *KRT15* | *KRT4* | *KRTAP10* | *KRTAP4* | *KRTAP5* | *LCE1F* |
| *LCE4A* | *LGALS9B* | *LIG1* | *LIMCH1* | *LMAN1* | *LMO1* | *LOC105374608* | *LOC107986229* | *LOR* | *LRP1B* | *LRP2* | *LTA* | *LURAP1L* | *LYN* | *LZTR1* |
| *M6PR* | *MAGI2* | *MAN1B1* | *MAP2K1* | *MAP2K2* | *MAP2K4* | *MAP3K1* | *MCCC2* | *MCHR2* | *MCL1* | *MCMDC2* | *MDM2* | *MDM4* | *MED12* | *MED13* |
| *MEF2B* | *MEN1* | *MET* | *MITF* | *MLH1* | *MLLT3* | *MPL* | *MRE11A* | *MSH2* | *MSH3* | *MSH6* | *MTHFR* | *MTOR* | *MUC2* | *MUC4* |
| *MUC6* | *MUTYH* | *MVK* | *MYB* | *MYC* | *MYCL* | *MYCN* | *MYD88* | *MYL1* | *MYOM1* | *NBEA* | *NBN* | *NCOA3* | *NCOA6* | *NCOR2* |
| *NEFH* | *NF1* | *NF2* | *NFE2L2* | *NFKBIA* | *NFXL1* | *NIPBL* | *NKX2-1* | *NOTCH1* | *NOTCH2* | *NOTCH3* | *NPM1* | *NQO1* | *NR1H2* | *NRAS* |
| *NRP2* | *NSD1* | *NTRK1* | *NTRK2* | *NTRK3* | *NUP155* | *NUP93* | *OPRK1* | *OR11H4* | *OR2B11* | *OR52D1* | *OR5K4* | *OR6C76* | *OR8I2* | *ORAI1* |
| *PAK3* | *PALB2* | *PARK2* | *PAX5* | *PBRM1* | *PCDH12* | *PDCD1LG2* | *PDE11A* | *PDE7A* | *PDGFRA* | *PDGFRB* | *PDK1* | *PHGR1* | *PIK3C2B* | *PIK3C3* |
| *PIK3CA* | *PIK3CB* | *PIK3CG* | *PIK3R1* | *PIK3R2* | *PIP4K2A* | *PKD1L2* | *PLCG2* | *PMS2* | *POLD1* | *POLE* | *POLI* | *POTEC* | *PPP2R1A* | *PPP6C* |
| *PRDM1* | *PREX2* | *PRKAR1A* | *PRKCH* | *PRKCI* | *PRKDC* | *PRKRA* | *PROSER3* | *PRPF19* | *PRSS8* | *PRX* | *PTCH1* | *PTEN* | *PTGS2* | *PTPN11* |
| *QKI* | *RAC1* | *RAD50* | *RAD51* | *RAF1* | *RALY* | *RANBP2* | *RARA* | *RB1* | *RBM10* | *RBM27* | *RBM5* | *RET* | *RETNLB* | *RFX3* |
| *RGPD3* | *RGS12* | *RHPN2* | *RIC8A* | *RICTOR* | *RIN3* | *RNF145* | *RNF213* | *RNF43* | *ROCK1* | *ROS1* | *RP1L1* | *RPL8* | *RPS12* | *RPS9* |
| *RPTN* | *RPTOR* | *RRM1* | *RSBN1L* | *RUNX1* | *RUNX1T1* | *SCAI* | *SCYL2* | *SDHA* | *SDHB* | *SDHC* | *SDHD* | *SEC31A* | *SELE* | *SELPLG* |
| *[SEMA3C](https://www.snpedia.com/index.php/Special:FormEdit/Gene/SEMA3C)* | *SEMA5B* | *SEPP1* | *SERPINA10* | *SETBP1* | *SETD2* | *SF3B1* | *SH3GL1* | *SI* | *SIK2* | *SKIDA1* | *SLAMF1* | *SLC11A2* | *SLC19A1* | *SLC22A2* |
| *SLC28A3* | *SLC29A1* | *SLC2A5* | *SLC35F5* | *SLC35G2* | *SLC36A2* | *SLC3A2* | *SLC6A18* | *SLCO1B3* | *SLIT2* | *SLK* | *SLX4* | *SMAD2* | *SMAD3* | *SMAD4* |
| *SMARCA4* | *SMARCB1* | *SMO* | *SNCAIP* | *SNX13* | *SOCS1* | *SOD2* | *SOX10* | *SOX11* | *SOX2* | *SOX9* | *SPATA3* | *SPEN* | *SPOP* | *SPTA1* |
| *SRBD1* | *SRC* | *SRD5A2* | *SRGAP3* | *SRPR* | *SRSF2* | *SSTR4* | *ST18* | *STAG2* | *STAT3* | *STAT4* | *STK11* | *STMN1* | *SUFU* | *SULT1A1* |
| *SVIL* | *SYK* | *TAF1* | *TAF1B* | *TBC1D23* | *TBK1* | *TBX3* | *TCERG1* | *TEAD2* | *TERC* | *TERT* | *TET2* | *TFAM* | *TGFBR2* | *THAP2* |
| *THAP3* | *THAP5* | *TMBIM4* | *TMEM106B* | *TMEM37* | *TMEM60* | *TMEM97* | *TNFAIP3* | *TNFAIP6* | *TNFRSF14* | *TOMM70* | *TOP1* | *TOP2A* | *TP53* | *TPMT* |
| *TRIM48* | *TRIM51* | *TRMT10C* | *TRRAP* | *TSC1* | *TSC2* | *TSHR* | *TTK* | *TTLL10* | *TVP23A* | *TXNDC2* | *TYMS* | *U2AF1* | *UBA7* | *UBE3C* |
| *UBE4A* | *UBR5* | *UGT1A1* | *UHRF1* | *ULK4* | *UMPS* | *UPF3A* | *USP35* | *USP36* | *VEGFA* | *VEZT* | *VHL* | *VIT* | *WDR37* | *WDR66* |
| *WDTC1* | *WISP3* | *WT1* | *XPC* | *XPO1* | *XRCC1* | *XRCC2* | *XYLT2* | *ZBTB2* | *ZFP37* | *ZFR2* | *ZNF217* | *ZNF365* | *ZNF429* | *ZNF462* |
| *ZNF479* | *ZNF516* | *ZNF527* | *ZNF605* | *ZNF703* | *ZNF717* | *ZNF776* | *ZNF814* | *ZNF844* |  |  |  |  |  |  |

**Table S3. Detailed clinical and genomic feature information for each enrolled gastric cancer patient.**

| **Patient ID** | **PD-L1 expression score**  **(CPS)** | **TMB score**  **(muts/Mb)** | **MSI score** | **Gender** | **Age** | ***HER2***  **(copies)** |
| --- | --- | --- | --- | --- | --- | --- |
| P001 | ~ 65 | 13.2 | 50 | Female | 76 | 2 |
| P002 | ~ 25 | 9.6 | 13.64 | Female | 78 | 5.5 |
| P003 | ~ 30 | 3.7 | 36.36 | Female | 63 | 2 |
| P004 | ~ 20 | 2.9 | 4.54 | Male | 60 | 2 |
| P005 | ~ 30 | 4.4 | 18.18 | Female | 75 | 3.5 |
| P006 | ~ 100 | 14.7 | 18.18 | Male | 57 | 2 |
| P007 | ~ 25 | 0.7 | 4.54 | Male | 64 | 2 |
| P008 | ~ 45 | 2.9 | 0 | Male | 70 | 2 |
| P009 | ~ 40 | 0.7 | 0 | Female | 55 | 2 |
| P010 | ~ 35 | 2.2 | 31.82 | Male | 60 | 2 |
| P011 | ~ 55 | 2.2 | 9.09 | Male | 54 | 2 |
| P012 | ~ 95 | 1.5 | 0 | Male | 55 | 2 |
| P013 | ~ 55 | 2.9 | 4.54 | Female | 48 | 2 |
| P014 | ~ 20 | 34.6 | 72.73 | Male | 75 | 2 |
| P015 | ~ 70 | 27.2 | 40.91 | Female | 61 | 2 |
| P016 | ~ 85 | 33.8 | 63.64 | Female | 73 | 2 |
| P017 | ~ 20 | 4.4 | 0 | Female | 54 | 2 |
| P018 | ~ 35 | 5.9 | 0 | Male | 67 | 2 |
| P019 | ~ 35 | 2.2 | 0 | Male | 64 | 2 |
| P020 | ~ 35 | 29.4 | 54.54 | Female | 76 | 2 |
| P021 | ~ 97 | 5.1 | 0 | Male | 69 | 2 |
| P022 | ~ 80 | 22.8 | 50 | Male | 69 | 2 |
| P023 | ~ 95 | 3.7 | 0 | Male | 54 | 2 |
| P024 | ~ 60 | 2.9 | 0 | Male | 59 | 2 |
| P025 | ~ 25 | 6.6 | 0 | Female | 49 | 2 |
| P026 | ~ 45 | 7.4 | 0 | Male | 62 | 2 |
| P027 | ~ 35 | 2.9 | 4.54 | Male | 53 | 2 |
| P028 | ~ 30 | 19.1 | 41 | Female | 77 | 2 |
| P029 | ~ 65 | 1.5 | 4.54 | Male | 72 | 2 |
| P030 | ~ 30 | 8.1 | 0 | Female | 54 | 2 |
| P031 | ~ 40 | 7.4 | 0 | Female | 70 | 2 |
| P032 | ~ 60 | 2.9 | 0 | Male | 72 | 2 |
| P033 | ~ 85 | 2.9 | 8.33 | Female | 63 | 2 |
| P034 | ~ 60 | 4.4 | 0 | Female | 53 | 2 |
| P035 | ~ 20 | 4.4 | 12.5 | Female | 62 | 2 |
| P036 | ~ 45 | 2.2 | 4.17 | Female | 54 | 2 |
| P037 | ~ 90 | 2.2 | 0 | Male | 58 | 2 |
| P038 | ~ 20 | 6.6 | 4.17 | Male | 55 | 2 |
| P039 | ~ 65 | 5.9 | 0 | Male | 51 | 2 |
| P040 | ~ 25 | 5.9 | 0 | Male | 68 | 2 |
| P041 | ~ 15 | 4.4 | 31.82 | Male | 76 | 2 |
| P042 | < 1 | 2.2 | 13.64 | Male | 56 | 4 |
| P043 | ~ 1-2 | 3.7 | 18.18 | Male | 66 | 2 |
| P044 | < 1 | 2.2 | 18.18 | Male | 30 | 34.5 |
| P045 | ~ 10 | 2.9 | 36.36 | Male | 78 | 2 |
| P046 | < 1 | 9.6 | 31.82 | Male | 61 | 2 |
| P047 | < 1 | 0.7 | 22.73 | Male | 62 | 2 |
| P048 | < 1 | 5.9 | 31.82 | Female | 55 | 2 |
| P049 | < 1 | 5.1 | 13.64 | Female | 41 | 2 |
| P050 | ~ 1-2 | 2.9 | 27.27 | Male | 60 | 2 |
| P051 | ~ 3-5 | 2.2 | 22.73 | Male | 75 | 2 |
| P052 | ~ 1-2 | 4.4 | 22.73 | Male | 83 | 2 |
| P053 | < 1 | 3.7 | 18.18 | Male | 75 | 2 |
| P054 | ~ 2 | 3.7 | 13.64 | Male | 60 | 2.5 |
| P055 | < 1 | 1.5 | 13.64 | Male | 54 | 6 |
| P056 | ~ 5 | 5.1 | 18.18 | Male | 71 | 34.5 |
| P057 | ~ 3 | 1.5 | 27.27 | Male | 68 | 2 |
| P058 | < 1 | 7.4 | 22.73 | Male | 67 | 2 |
| P059 | < 1 | 1.5 | 27.27 | Male | 69 | 2 |
| P060 | < 1 | 3.7 | 22.73 | Female | 56 | 2 |
| P061 | < 1 | 0.7 | 13.64 | Male | 64 | 2 |
| P062 | < 1 | 25.7 | 72.73 | Female | 55 | 2 |
| P063 | < 1 | 3.7 | 22.73 | Female | 39 | 2 |
| P064 | < 1 | 19.9 | 27.27 | Male | 72 | 5.5 |
| P065 | < 1 | 0.7 | 36.36 | Male | 68 | 2 |
| P066 | < 1 | 2.9 | 36.36 | Female | 70 | 2 |
| P067 | < 1 | 2.2 | 12.5 | Male | 51 | 2 |
| P068 | < 1 | 5.9 | 27.27 | Male | 51 | 2 |
| P069 | < 1 | 2.2 | 22.73 | Female | 31 | 2 |
| P070 | < 1 | 1.5 | 22.73 | Female | 40 | 2 |
| P071 | < 1 | 4.4 | 13.64 | Female | 52 | 2 |
| P072 | ~ 10 | 1.5 | 18.18 | Male | 28 | 2 |
| P073 | < 1 | 2.9 | 13.64 | Male | 67 | 4 |
| P074 | < 1 | 4.4 | 22.73 | Male | 28 | 5 |
| P075 | < 1 | 5.9 | 27.27 | Male | 49 | 2 |
| P076 | ~ 2 | 2.2 | 18.18 | Male | 61 | 2 |
| P077 | < 1 | 2.9 | 13.64 | Male | 56 | 2 |
| P078 | < 1 | 2.9 | 18.18 | Male | 65 | 2 |
| P079 | < 1 | 4.4 | 11.77 | Male | 66 | 2 |
| P080 | ~ 8 | 2.9 | 13.64 | Male | 34 | 2 |
| P081 | ~ 2 | 9.6 | 9.09 | Male | 83 | 2 |
| P082 | ~ 10 | 6.6 | 9.09 | Male | 81 | 2 |
| P083 | < 1 | 2.9 | 18.18 | Female | 62 | 2 |
| P084 | < 1 | 5.1 | 18.18 | Female | 67 | 2 |
| P085 | < 1 | 0.7 | 22.73 | Female | 36 | 2 |
| P086 | < 1 | 1.5 | 5.88 | Female | 79 | 2 |
| P087 | ~ 3 | 1.5 | 13.64 | Male | 74 | 2 |
| P088 | < 1 | 2.2 | 18.18 | Male | 67 | 2 |
| P089 | < 1 | 1.5 | 27.27 | Male | 55 | 2 |
| P090 | < 1 | 6.6 | 13.64 | Female | 66 | 2 |
| P091 | < 1 | 8.1 | 22.73 | Female | 67 | 2 |
| P092 | < 1 | 20.6 | 11.11 | Male | 71 | 2 |
| P093 | ~ 3 | 1.5 | 4.54 | Male | 72 | 2 |
| P094 | < 1 | 3.7 | 27.27 | Male | 62 | 2 |
| P095 | < 1 | 13.2 | 35.29 | Female | 73 | 2 |
| P096 | ~ 2 | 0.7 | 18.18 | Male | 66 | 2 |
| P097 | ~ 3 | 2.2 | 11.77 | Female | 67 | 23 |
| P098 | < 1 | 6.6 | 13.64 | Male | 66 | 2 |
| P099 | < 1 | 8.8 | 9.09 | Male | 56 | 12.5 |
| P100 | < 1 | 4.4 | 13.64 | Male | 70 | 2 |
| P101 | ~ 8 | 1.5 | 33.33 | Female | 70 | 2 |
| P102 | < 1 | 1.5 | 13.64 | Female | 51 | 2 |
| P103 | < 1 | 9.6 | 4.54 | Male | 55 | 6 |
| P104 | < 1 | 2.9 | 5.88 | Male | 74 | 2 |
| P105 | < 1 | 32.4 | 63.64 | Male | 74 | 2 |
| P106 | < 1 | 4.4 | 9.09 | Female | 78 | 2 |
| P107 | ~ 3 | 15.4 | 47.62 | Male | 67 | 2 |
| P108 | < 1 | 8.1 | 4.54 | Male | 62 | 2 |
| P109 | < 1 | 2.2 | 0 | Male | 58 | 2 |
| P110 | < 1 | 5.1 | 9.09 | Male | 62 | 3.5 |
| P111 | < 1 | 3.7 | 4.54 | Male | 75 | 12.5 |
| P112 | < 1 | 8.1 | 4.76 | Female | 62 | 2 |
| P113 | ~ 5 | 1.5 | 0 | Male | 72 | 2 |
| P114 | ~ 2 | 2.2 | 0 | Male | 53 | 2 |
| P115 | < 1 | 8.8 | 4.54 | Male | 60 | 2 |
| P116 | ~ 1-2 | 2.2 | 4.54 | Female | 37 | 2 |
| P117 | < 1 | 1.5 | 0 | Male | 69 | 3.5 |
| P118 | ~ 1-2 | 1.5 | 21.05 | Male | 74 | 2 |
| P119 | < 1 | 2.2 | 13.64 | Male | 39 | 2 |
| P120 | < 1 | 2.2 | 11.77 | Male | 65 | 3.5 |
| P121 | < 1 | 3.7 | 4.54 | Male | 88 | 2 |
| P122 | ~ 15 | 1.5 | 4.54 | Male | 66 | 2.5 |
| P123 | < 1 | 3.7 | 0 | Male | 62 | 2 |
| P124 | < 1 | 4.4 | 9.09 | Female | 49 | 2 |
| P125 | < 1 | 2.2 | 4.54 | Male | 63 | 2 |
| P126 | ~ 5 | 36 | 50 | Female | 71 | 2 |
| P127 | < 1 | 2.9 | 0 | Male | 55 | 2 |
| P128 | < 1 | 0.7 | 4.54 | Female | 67 | 2 |
| P129 | ~ 5 | 1.5 | 4.54 | Male | 53 | 16 |
| P130 | < 1 | 2.9 | 9.09 | Male | 67 | 2 |
| P131 | ~ 10 | 25.7 | 59.09 | Male | 67 | 2 |
| P132 | < 1 | 0.7 | 0 | Male | 56 | 2 |
| P133 | < 1 | 4.4 | 9.09 | Male | 73 | 2 |
| P134 | ~ 1-2 | 6.6 | 22.73 | Male | 67 | 2 |
| P135 | < 1 | 0.7 | 0 | Male | 58 | 2 |
| P136 | < 1 | 1.5 | 4.54 | Male | 52 | 2 |
| P137 | ~ 1-2 | 2.9 | 13.64 | Male | 55 | 2 |
| P138 | ~ 3 | 5.9 | 9.09 | Male | 69 | 2 |
| P139 | < 1 | 34.6 | 54.54 | Female | 71 | 2 |
| P140 | < 1 | 6.6 | 9.09 | Male | 68 | 2 |
| P141 | < 1 | 4.4 | 0 | Male | 41 | 2 |
| P142 | < 1 | 5.1 | 9.09 | Male | 62 | 2 |
| P143 | < 1 | 5.9 | 18.18 | Male | 52 | 2 |
| P144 | ~ 5 | 28.7 | 50 | Male | 79 | 2 |
| P145 | < 1 | 1.5 | 0 | Male | 39 | 2 |
| P146 | < 1 | 2.9 | 4.54 | Male | 55 | 2 |
| P147 | ~ 5 | 5.9 | 0 | Male | 69 | 2 |
| P148 | < 1 | 3.7 | 15 | Male | 55 | 2 |
| P149 | < 1 | 1.5 | 4.54 | Male | 57 | 2 |
| P150 | < 1 | 0.7 | 9.09 | Male | 55 | 3.5 |
| P151 | ~ 2 | 2.9 | 13.64 | Female | 68 | 2 |
| P152 | < 1 | 5.9 | 22.73 | Female | 52 | 2 |
| P153 | ~ 10 | 2.9 | 9.09 | Female | 69 | 2 |
| P154 | < 1 | 3.7 | 18.18 | Female | 67 | 2 |
| P155 | ~ 1-2 | 2.9 | 9.09 | Male | 69 | 2 |
| P156 | < 1 | 2.9 | 18.18 | Male | 66 | 3.5 |
| P157 | ~ 5 | 2.9 | 13.64 | Male | 60 | 2 |
| P158 | < 1 | 27.9 | 77.27 | Male | 77 | 2 |
| P159 | ~ 8 | 2.9 | 13.64 | Male | 69 | 2 |
| P160 | ~ 2 | 33.1 | 63.64 | Female | 70 | 2 |
| P161 | < 1 | 9.6 | 9.09 | Male | 66 | 2 |
| P162 | ~ 10 | 1.5 | 0 | Female | 63 | 4.5 |
| P163 | ~ 6 | 40.4 | 77.27 | Male | 61 | 2 |
| P164 | ~ 5 | 4.4 | 4.54 | Male | 66 | 2 |
| P165 | < 1 | 3.7 | 9.09 | Male | 68 | 2 |
| P166 | < 1 | 2.9 | 4.54 | Male | 65 | 2 |
| P167 | ~ 2 | 2.9 | 4.54 | Male | 52 | 10 |
| P168 | ~ 15 | 2.2 | 0 | Male | 73 | 2 |
| P169 | < 1 | 2.9 | 4.54 | Female | 64 | 2 |
| P170 | ~ 8 | 1.5 | 4.76 | Male | 55 | 2 |
| P171 | ~ 6 | 2.2 | 27.27 | Female | 57 | 2 |
| P172 | < 1 | 6.6 | 0 | Male | 59 | 2 |
| P173 | ~ 5 | 1.5 | 9.09 | Male | 70 | 3.5 |
| P174 | ~ 1-2 | 0.7 | 0 | Female | 74 | 2 |
| P175 | ~ 3 | 2.9 | 0 | Female | 69 | 2 |
| P176 | < 1 | 2.2 | 0 | Male | 56 | 2 |
| P177 | ~ 15 | 1.5 | 4.54 | Male | 66 | 2 |
| P178 | ~ 1-2 | 0.7 | 0 | Male | 55 | 2 |
| P179 | < 1 | 2.9 | 4.54 | Male | 72 | 2 |
| P180 | < 1 | 1.5 | 0 | Male | 69 | 2 |
| P181 | ~ 6 | 1.5 | 4.54 | Male | 57 | 2 |
| P182 | < 1 | 0.7 | 0 | Male | 78 | 2 |
| P183 | < 1 | 2.2 | 0 | Male | 65 | 3.5 |
| P184 | ~ 8 | 4.4 | 4.54 | Male | 75 | 2 |
| P185 | < 1 | 2.2 | 0 | Male | 57 | 2 |
| P186 | ~ 10 | 1.5 | 0 | Male | 71 | 2 |
| P187 | ~ 8 | 5.9 | 0 | Female | 64 | 2 |
| P188 | < 1 | 5.1 | 9.09 | Female | 73 | 2 |
| P189 | < 1 | 3.7 | 0 | Female | 62 | 2 |
| P190 | < 1 | 0.7 | 13.64 | Female | 58 | 2 |
| P191 | ~ 8 | 2.9 | 9.09 | Male | 48 | 2 |
| P192 | ~ 10 | 13.2 | 4.54 | Male | 65 | 2 |
| P193 | < 1 | 2.9 | 9.09 | Male | 76 | 2 |
| P194 | < 1 | 2.2 | 4.54 | Male | 48 | 2 |
| P195 | ~ 10 | 2.9 | 4.54 | Male | 66 | 2 |
| P196 | ~ 10 | 2.2 | 0 | Male | 66 | 2 |
| P197 | ~ 2 | 5.1 | 0 | Female | 69 | 2 |
| P198 | < 1 | 5.1 | 13.64 | Male | 49 | 2 |
| P199 | ~ 5 | 6.6 | 0 | Male | 77 | 2 |
| P200 | ~ 2 | 7.4 | 0 | Male | 69 | 3.5 |
| P201 | ~ 12 | 40.4 | 72.73 | Female | 68 | 2 |
| P202 | ~ 12 | 2.9 | 0 | Male | 69 | 2 |
| P203 | ~ 3 | 2.2 | 0 | Male | 47 | 2 |
| P204 | ~ 5 | 3.7 | 0 | Male | 61 | 2 |
| P205 | < 1 | 5.1 | 0 | Male | 70 | 2 |
| P206 | < 1 | 15.4 | 9.09 | Female | 41 | 2 |
| P207 | < 1 | 2.2 | 0 | Female | 64 | 2 |
| P208 | ~ 3 | 2.9 | 4.54 | Male | 57 | 2 |
| P209 | < 1 | 3.7 | 9.09 | Male | 72 | 2 |
| P210 | < 1 | 1.5 | 9.09 | Female | 71 | 2 |
| P211 | < 1 | 5.9 | 9.09 | Male | 67 | 2 |
| P212 | < 1 | 4.4 | 4.54 | Female | 64 | 2 |
| P213 | < 1 | 5.1 | 0 | Male | 74 | 2 |
| P214 | < 1 | 3.7 | 0 | Female | 46 | 2 |
| P215 | < 1 | 1.5 | 9.09 | Male | 64 | 2 |
| P216 | ~ 5 | 5.9 | 4.54 | Male | 68 | 2 |
| P217 | < 1 | 30.9 | 81.82 | Female | 67 | 2 |
| P218 | < 1 | 5.1 | 4.54 | Male | 55 | 2 |
| P219 | ~ 8 | 3.7 | 0 | Male | 66 | 2 |
| P220 | < 1 | 12.5 | 0 | Male | 72 | 2 |
| P221 | < 1 | 8.1 | 0 | Male | 58 | 2 |
| P222 | < 1 | 2.2 | 4.54 | Male | 56 | 2 |
| P223 | ~ 12 | 4.4 | 0 | Female | 54 | 2 |
| P224 | < 1 | 2.9 | 4.54 | Male | 69 | 2 |
| P225 | < 1 | 0.7 | 0 | Female | 54 | 2 |
| P226 | < 1 | 0.7 | 0 | Female | 48 | 4.5 |
| P227 | < 1 | 1.5 | 4.54 | Male | 71 | 2 |
| P228 | < 1 | 2.2 | 0 | Male | 69 | 2 |
| P229 | < 1 | 3.7 | 0 | Male | 74 | 3.5 |
| P230 | ~ 5 | 0.7 | 4.54 | Male | 48 | 2 |
| P231 | ~ 3 | 3.7 | 0 | Male | 53 | 2 |
| P232 | < 1 | 3.7 | 0 | Female | 51 | 2 |
| P233 | < 1 | 2.9 | 0 | Male | 36 | 2 |
| P234 | < 1 | 0.7 | 0 | Male | 61 | 2 |
| P235 | ~ 5 | 2.2 | 0 | Female | 59 | 2 |
| P236 | ~ 2 | 4.4 | 0 | Male | 65 | 4 |
| P237 | < 1 | 2.2 | 13.64 | Male | 78 | 2 |
| P238 | ~ 15 | 0.7 | 4.54 | Male | 58 | 2 |
| P239 | < 1 | 2.9 | 4.54 | Male | 61 | 2 |
| P240 | < 1 | 3.7 | 4.54 | Male | 60 | 2 |
| P241 | ~ 5 | 1.5 | 0 | Male | 72 | 2 |
| P242 | < 1 | 6.6 | 0 | Male | 69 | 3.5 |
| P243 | < 1 | 4.4 | 0 | Female | 76 | 2 |
| P244 | ~ 2 | 2.9 | 0 | Female | 71 | 2 |
| P245 | ~ 5 | 3.7 | 0 | Female | 69 | 2 |
| P246 | ~ 5 | 1.5 | 0 | Male | 61 | 2 |
| P247 | ~ 5 | 0.7 | 0 | Male | 69 | 2 |
| P248 | < 1 | 0.7 | 4.54 | Male | 76 | 2 |
| P249 | < 1 | 6.6 | 0 | Female | 40 | 2 |
| P250 | < 1 | 10.3 | 4.54 | Male | 56 | 2 |
| P251 | ~ 8 | 1.5 | 0 | Male | 77 | 2 |
| P252 | < 1 | 4.4 | 0 | Female | 60 | 2 |
| P253 | < 1 | 3.7 | 4.54 | Male | 55 | 2 |
| P254 | ~ 5 | 8.1 | 0 | Female | 61 | 2 |
| P255 | ~ 5 | 4.9 | 4.54 | Male | 72 | 2 |
| P256 | ~ 8 | 2.2 | 0 | Male | 77 | 2 |
| P257 | ~ 7 | 4.4 | 0 | Male | 71 | 2 |
| P258 | ~ 8 | 2.2 | 4.54 | Male | 70 | 2 |
| P259 | ~ 8 | 5.1 | 4.54 | Female | 73 | 2 |
| P260 | ~ 8 | 5.9 | 4.54 | Male | 71 | 2 |
| P261 | < 1 | 2.9 | 0 | Male | 62 | 2 |
| P262 | < 1 | 5.1 | 0 | Male | 72 | 2 |
| P263 | < 1 | 2.2 | 0 | Male | 68 | 2 |
| P264 | ~ 3 | 2.9 | 0 | Male | 80 | 2 |
| P265 | < 1 | 4.4 | 4.54 | Male | 67 | 2 |
| P266 | ~ 15 | 5.1 | 0 | Female | 64 | 2 |
| P267 | ~ 3 | 8.1 | 0 | Male | 74 | 2 |
| P268 | ~ 8 | 0.7 | 0 | Male | 66 | 3.5 |
| P269 | ~ 5 | 4.4 | 9.09 | Male | 64 | 2 |
| P270 | ~ 3 | 6.6 | 0 | Male | 70 | 2 |
| P271 | < 1 | 8.1 | 0 | Male | 71 | 3.5 |
| P272 | ~ 3 | 4.4 | 4.54 | Female | 56 | 3.5 |
| P273 | ~ 5 | 2.9 | 4.54 | Female | 60 | 2 |
| P274 | ~ 2 | 2.2 | 0 | Male | 60 | 2 |
| P275 | ~ 8 | 6.6 | 0 | Male | 72 | 2 |
| P276 | ~ 2 | 4.4 | 4.54 | Male | 42 | 2 |
| P277 | ~ 3 | 3.7 | 0 | Male | 69 | 3.5 |
| P278 | < 1 | 6.6 | 0 | Male | 68 | 5.5 |
| P279 | ~ 12 | 2.2 | 0 | Male | 63 | 2 |
| P280 | ~ 5 | 12.5 | 41.67 | Male | 77 | 2 |
| P281 | < 1 | 10.3 | 54.17 | Male | 56 | 2 |
| P282 | ~ 8 | 13.2 | 46.15 | Female | 46 | 2 |
| P283 | ~ 3 | 5.9 | 9.09 | Female | 80 | 2 |
| P284 | ~ 15 | 2.2 | 0 | Male | 74 | 2 |
| P285 | ~ 7 | 2.2 | 0 | Male | 68 | 2 |
| P286 | < 1 | 4.4 | 0 | Male | 54 | 2 |
| P287 | ~ 5 | 4.4 | 0 | Male | 78 | 2 |
| P288 | < 1 | 5.1 | 0 | Male | 49 | 2 |
| P289 | ~ 3 | 3.7 | 0 | Male | 69 | 2 |
| P290 | ~ 7 | 5.1 | 4.54 | Male | 69 | 2 |
| P291 | < 1 | 6.6 | 0 | Male | 64 | 3 |
| P292 | ~ 2 | 1.5 | 0 | Male | 43 | 2 |
| P293 | ~ 8 | 1.5 | 0 | Female | 52 | 2 |
| P294 | < 1 | 2.9 | 4.54 | Male | 62 | 2 |
| P295 | < 1 | 5.1 | 0 | Female | 67 | 3.5 |
| P296 | ~ 2 | 4.4 | 0 | Male | 58 | 2 |
| P297 | < 1 | 1.5 | 0 | Female | 49 | 2 |
| P298 | ~ 2 | 4.4 | 0 | Male | 47 | 2 |
| P299 | ~ 10 | 5.1 | 4.54 | Male | 59 | 2 |
| P300 | ~ 3 | 7.4 | 4.54 | Male | 60 | 2 |
| P301 | < 1 | 3.7 | 0 | Male | 53 | 2 |
| P302 | < 1 | 2.2 | 0 | Male | 67 | 2 |
| P303 | < 1 | 3.7 | 9.09 | Male | 76 | 2 |
| P304 | < 1 | 4.4 | 0 | Male | 74 | 2 |
| P305 | < 1 | 2.9 | 4.54 | Male | 68 | 2 |
| P306 | < 1 | 5.1 | 0 | Male | 59 | 5.5 |
| P307 | ~ 2 | 3.7 | 9.09 | Male | 55 | 2 |
| P308 | < 1 | 0.7 | 4.54 | Male | 53 | 2 |
| P309 | ~ 2 | 14 | 54.54 | Male | 58 | 2 |
| P310 | < 1 | 24.3 | 68.18 | Female | 72 | 2 |
| P311 | < 1 | 7.4 | 0 | Male | 65 | 2 |
| P312 | ~ 5 | 6.6 | 0 | Male | 83 | 2 |
| P313 | < 1 | 5.1 | 4.54 | Male | 54 | 2 |
| P314 | < 1 | 39 | 54.54 | Female | 69 | 2 |
| P315 | < 1 | 8.8 | 40.91 | Female | 60 | 2 |
| P316 | ~ 5 | 2.9 | 0 | Male | 57 | 2 |
| P317 | < 1 | 4.4 | 0 | Male | 64 | 2 |
| P318 | < 1 | 2.2 | 4.54 | Male | 60 | 2 |
| P319 | ~ 2 | 36 | 77.27 | Male | 60 | 2 |
| P320 | ~ 2 | 3.7 | 4.54 | Female | 73 | 2 |
| P321 | ~ 5 | 4.4 | 0 | Female | 46 | 2 |
| P322 | < 1 | 4.4 | 0 | Male | 61 | 2 |
| P323 | < 1 | 2.9 | 4.54 | Female | 44 | 2 |
| P324 | ~ 2 | 1.5 | 0 | Female | 47 | 2 |
| P325 | ~ 5 | 5.1 | 4.17 | Male | 74 | 2 |
| P326 | < 1 | 1.5 | 4.17 | Male | 53 | 2 |
| P327 | < 1 | 1.5 | 0 | Male | 85 | 2 |
| P328 | < 1 | 3.7 | 8.33 | Male | 77 | 2 |
| P329 | < 1 | 2.2 | 4.17 | Male | 64 | 2 |
| P330 | ~ 2 | 5.9 | 20.83 | Female | 57 | 2 |
| P331 | ~ 7 | 3.7 | 4.17 | Male | 80 | 2 |
| P332 | < 1 | 3.7 | 4.17 | Male | 57 | 2 |
| P333 | ~ 5 | 8.1 | 12.5 | Male | 71 | 5 |
| P334 | ~ 2 | 7.4 | 0 | Female | 74 | 2 |
| P335 | ~ 1-2 | 4.4 | 8.33 | Male | 52 | 3.5 |
| P336 | < 1 | 2.2 | 4.17 | Male | 66 | 2 |
| P337 | < 1 | 0.7 | 0 | Male | 49 | 2 |
| P338 | < 1 | 3.7 | 4.17 | Female | 70 | 2 |
| P339 | < 1 | 5.9 | 0 | Male | 71 | 2 |
| P340 | < 1 | 2.9 | 4.17 | Male | 75 | 2 |
| P341 | < 1 | 4.4 | 4.17 | Male | 68 | 2 |
| P342 | < 1 | 1.5 | 4.17 | Male | 59 | 2 |
| P343 | ~ 2 | 4.4 | 8.33 | Male | 57 | 2 |
| P344 | ~ 15 | 4.4 | 0 | Male | 79 | 2 |
| P345 | < 1 | 4.4 | 8.33 | Female | 47 | 2 |
| P346 | < 1 | 2.9 | 4.17 | Female | 49 | 2 |
| P347 | ~ 10 | 30.1 | 79.17 | Male | 70 | 2 |
| P348 | < 1 | 3.7 | 4.17 | Female | 55 | 2 |
| P349 | ~ 3 | 2.2 | 0 | Male | 80 | 2 |
| P350 | < 1 | 2.2 | 0 | Male | 62 | 2 |
| P351 | ~ 5 | 0.7 | 4.17 | Female | 71 | 2 |
| P352 | ~ 3 | 4.4 | 8.33 | Male | 49 | 2 |
| P353 | < 1 | 0.7 | 4.17 | Male | 48 | 2 |
| P354 | < 1 | 4.4 | 0 | Male | 59 | 26 |
| P355 | ~ 5 | 2.9 | 4.17 | Male | 71 | 2 |
| P356 | ~ 3 | 8.8 | 4.17 | Female | 72 | 2 |
| P357 | ~ 5 | 3.7 | 0 | Male | 54 | 2 |
| P358 | ~ 5 | 6.6 | 0 | Male | 57 | 2 |
| P359 | < 1 | 5.9 | 0 | Male | 60 | 2 |
| P360 | < 1 | 5.9 | 12.5 | Male | 73 | 2.5 |
| P361 | ~ 7 | 5.1 | 4.17 | Male | 68 | 2 |
| P362 | < 1 | 6.6 | 8.33 | Male | 73 | 4 |
| P363 | ~ 5 | 39.7 | 62.5 | Male | 70 | 2 |
| P364 | ~ 2 | 3.7 | 0 | Male | 68 | 9.5 |
| P365 | < 1 | 5.9 | 12.5 | Male | 60 | 2 |
| P366 | < 1 | 2.2 | 4.17 | Male | 69 | 2 |
| P367 | ~ 3 | 1.5 | 4.17 | Female | 47 | 2 |
| P368 | < 1 | 5.1 | 4.17 | Female | 82 | 2 |
| P369 | < 1 | 9.6 | 4.17 | Female | 52 | 2 |
| P370 | < 1 | 0.7 | 4.17 | Female | 70 | 2 |
| P371 | < 1 | 0.7 | 0 | Male | 54 | 2 |
| P372 | < 1 | 5.1 | 4.17 | Female | 71 | 2 |
| P373 | ~ 5 | 2.2 | 4.17 | Male | 46 | 2 |
| P374 | < 1 | 3.7 | 4.17 | Male | 57 | 2 |
| P375 | < 1 | 5.9 | 12.5 | Male | 52 | 2 |
| P376 | < 1 | 6.6 | 20.83 | Female | 69 | 2 |
| P377 | < 1 | 4.4 | 0 | Male | 61 | 2 |
| P378 | ~ 2 | 5.9 | 8.33 | Female | 62 | 2 |
| P379 | < 1 | 0.7 | 8.33 | Male | 54 | 2 |
| P380 | < 1 | 8.1 | 4.17 | Male | 74 | 2 |
| P381 | ~ 8 | 8.1 | 4.17 | Male | 66 | 2 |
| P382 | ~ 3 | 5.1 | 4.17 | Male | 60 | 2 |
| P383 | < 1 | 25 | 79.17 | Male | 66 | 2 |
| P384 | ~ 3 | 2.9 | 4.17 | Male | 64 | 15 |
| P385 | ~ 10 | 2.9 | 8.33 | Female | 79 | 2 |
| P386 | < 1 | 3.7 | 8.33 | Male | 52 | 2 |
| P387 | < 1 | 5.9 | 8.33 | Male | 61 | 2 |
| P388 | < 1 | 4.4 | 8.33 | Female | 66 | 2 |
| P389 | ~ 2 | 12.5 | 8.33 | Male | 70 | 2 |
| P390 | < 1 | 3.7 | 0 | Male | 60 | 2 |

**Table S4. Clinical and molecular characteristics stratified by *HER2* status based on NGS.**

| **HER2 NGS (copies ≥3.0)** | | | | | |
| --- | --- | --- | --- | --- | --- |
|  | **[ALL]**  **N=390** | **Negative**  **N=348** | **Positive**  **N=42** | **OR** | p.overalla |
| **Gender** |  |  |  |  | 0.115 |
| Female | 110 (28.2%) | 103 (29.6%) | 7 (16.7%) | Ref. |  |
| Male | 280 (71.8%) | 245 (70.4%) | 35(83.3%) | 2.06 [0.93;5.26] |  |
| **Ageatdiagnosisinyears** |  |  |  |  | 1.00 |
| 60-(<60years) | 134 (34.4%) | 120 (34.5%) | 14 (33.3%) | Ref. |  |
| 60+(≥60years) | 256 (65.6%) | 228 (65.5%) | 28 (66.7%) | 1.05 [0.54;2.13] |  |
| **TP53_Status** |  |  |  |  | 0.003 |
| Mutated | 228 (58.5%) | 194 (55.7%) | 34 (81.0%) | Ref. |  |
| Wild | 162 (41.5%) | 154 (44.3%) | 8 (19.0%) | 0.30 [0.13;0.64] |  |
| **TMB_Status** |  |  |  |  | 0.102 |
| TMB-H(≥10muts/Mb) | 38 (9.74%) | 37 (10.6%) | 1 (2.38%) | Ref. |  |
| TMB-L(<10muts/Mb) | 352 (90.3%) | 311 (89.4%) | 41 (97.6%) | 4.28 [0.89;102] |  |
| **MSI_Status** |  |  |  |  | 0.059 |
| MSI-H(≥40%) | 30 (7.69%) | 30 (8.62%) | 0 (0.00%) | Ref. |  |
| NonMSI-H(<40%) | 360 (92.3%) | 318 (91.4%) | 42 (100%) | [.;.] |  |
| **HER2 NGS (copies≥3.5)** | | | | | |
|  | **[ALL]**  **N=390** | **Negative**  **N=349** | **Positive**  **N=41** | **OR** | p.overalla |
| **Gender** |  |  |  |  | 0.136 |
| Female | 110 (28.2%) | 10 (29.5%) | 7(17.1%) | Ref. |  |
| Male | 280 (71.8%) | 246 (70.5%) | 34 (82.9%) | 2.00 [0.90;5.10] |  |
| **Ageatdiagnosisinyears** |  |  |  |  | 1.000 |
| 60-(<60years) | 134 (34.4%) | 120 (34.4%) | 14 (34.1%) | Ref. |  |
| 60+(≥60years) | 256 (65.6%) | 229 (65.6%) | 27 (65.9%) | 1.01 [0.51;2.05] |  |
| **TP53_Status** |  |  |  |  | 0.004 |
| Mutated | 228 (58.5%) | 195 (55.9%) | 33 (80.5%) | Ref. |  |
| Wild | 162 (41.5%) | 154 (44.1%) | 8 (19.5%) | 0.31 [0.13;0.67] |  |
| **TMB_Status** |  |  |  |  | 0.158 |
| TMB-H(≥10muts/Mb) | 38 (9.74%) | 37 (10.6%) | 1 (2.44%) | Ref. |  |
| TMB-L(<10muts/Mb) | 352 (90.3%) | 312 (89.4%) | 40 (97.6%) | 4.16 [0.87;99.3] |  |
| **MSI_Status** |  |  |  |  | 0.058 |
| MSI-H(≥40%) | 30 (7.69%) | 30 (8.60%) | 0 (0.00%) | Ref. |  |
| NonMSI-H(<40%) | 360 (92.3%) | 319 (91.4%) | 41 (100%) | [.;.] |  |
| **HER2 NGS (copies≥4.0)** | | | | | |
|  | **[ALL]**  **N=390** | **Negative**  **N=366** | **Positive**  **N=24** | **OR** | p.overalla |
| **Gender** |  |  |  |  | 0.288 |
| Female | 110 (28.2%) | 106 (29.0%) | 4 (16.7%) | Ref. |  |
| Male | 280 (71.8%) | 260 (71.0%) | 20 (83.3%) | 1.98 [0.72;7.10] |  |
| **Ageatdiagnosisinyears** |  |  |  |  | 0.317 |
| 60-(<60years) | 134 (34.4%) | 123 (33.6%) | 11 (45.8%) | Ref. |  |
| 60+(≥60years) | 256 (65.6%) | 243 (66.4%) | 13 (54.2%) | 0.60 [0.26;1.41] |  |
| **TP53_Status** |  |  |  |  | 0.056 |
| Mutated | 228 (58.5%) | 209 (57.1%) | 19 (79.2%) | Ref. |  |
| Wild | 162 (41.5%) | 157 (42.9%) | 5 (20.8%) | 0.36 [0.11;0.92] |  |
| **TMB_Status** |  |  |  |  | 0.493 |
| TMB-H(≥10muts/Mb) | 38 (9.74%) | 37 (10.1%) | 1 (4.17%) | Ref. |  |
| TMB-L(<10muts/Mb) | 352 (90.3%) | 329 (89.9%) | 23 (95.8%) | 2.28 [0.46;55.1] |  |
| **MSI_Status** |  |  |  |  | 0.240 |
| MSI-H(≥40%) | 30 (7.69%) | 30 (8.20%) | 0 (0.00%) | Ref. |  |
| NonMSI-H(<40%) | 360 (92.3%) | 336 (91.8%) | 24 (100%) | [.;.] |  |
| **HER2 NGS (copies≥5.0)** | | | | | |
|  | **[ALL]**  **N=390** | **Negative**  **N=372** | **Positive**  **N=18** | **OR** | p.overalla |
| **Gender** |  |  |  |  | 0.167 |
| Female | 110 (28.2%) | 108 (29.0%) | 2 (11.1%) | Ref. |  |
| Male | 280 (71.8%) | 264 (71.0%) | 16 (88.9%) | 3.07 [0.84;21.3] |  |
| **Ageatdiagnosisinyears** |  |  |  |  | 0.239 |
| 60-(<60years) | 134 (34.4%) | 125 (33.6%) | 9 (50.0%) | Ref. |  |
| 60+(≥60years) | 256 (65.6%) | 247 (66.4%) | 9 (50.0%) | 0.51 [0.19;1.35] |  |
| **TP53_Status** |  |  |  |  | 0.003 |
| Mutated | 228 (58.5%) | 211 (56.7%) | 17 (94.4%) | Ref. |  |
| Wild | 162 (41.5%) | 161 (43.3%) | 1 (5.56%) | 0.09 [0.00;0.43] |  |
| **TMB_Status** |  |  |  |  | 1.000 |
| TMB-H(≥10muts/Mb) | 38 (9.74%) | 37 (9.95%) | 1 (5.56%) | Ref. |  |
| TMB-L(<10muts/Mb) | 352 (90.3%) | 335 (90.1%) | 17 (94.4%) | 1.66 [0.32;40.6] |  |
| **MSI_Status** |  |  |  |  | 0.381 |
| MSI-H(≥40%) | 30 (7.69%) | 30 (8.06%) | 0 (0.00%) | Ref. |  |
| NonMSI-H(<40%) | 360 (92.3%) | 342 (91.9%) | 18 (100%) | [.;.] |  |
| **HER2 NGS (copies≥6.0)** | | | | | |
|  | **[ALL]**  **N=390** | **Negative**  **N=378** | **Positive**  **N=12** | **OR** | p.overalla |
| **Gender** |  |  |  |  | 0.192 |
| Female | 110 (28.2%) | 109 (28.8%) | 1 (8.33%) | Ref. |  |
| Male | 280 (71.8%) | 269 (71.2%) | 11 (91.7%) | 3.94 [0.75;97.8] |  |
| **Ageatdiagnosisinyears** |  |  |  |  | 0.118 |
| 60-(<60years) | 134 (34.4%) | 127 (33.6%) | 7 (58.3%) | Ref. |  |
| 60+(≥60years) | 256 (65.6%) | 251 (66.4%) | 5 (41.7%) | 0.37 [0.10;1.19] |  |
| **TP53_Status** |  |  |  |  | 0.017 |
| Mutated | 228 (58.5%) | 217 (57.4%) | 11 (91.7%) | Ref. |  |
| Wild | 162 (41.5%) | 161 (42.6%) | 1 (8.33%) | 0.14 [0.01;0.73] |  |
| **TMB_Status** |  |  |  |  | 0.616 |
| TMB-H(≥10muts/Mb) | 38 (9.74%) | 38 (10.1%) | 0 (0.00%) | Ref. |  |
| TMB-L(<10muts/Mb) | 352 (90.3%) | 340 (89.9%) | 12 (100%) | [.;.] |  |
| **MSI_Status** |  |  |  |  | 0.611 |
| MSI-H(≥40%) | 30 (7.69%) | 30 (7.94%) | 0 (0.00%) | Ref. |  |
| NonMSI-H(<40%) | 360 (92.3%) | 348 (92.1%) | 12 (100%) | [.;.] |  |

**Table S5. Distribution characteristics of PD-L1 expression grouped by HER2 status based on NGS.**

| **HER2 NGS (copies ≥2.5)** | | | | | |
| --- | --- | --- | --- | --- | --- |
|  | **[ALL]**  **N=390** | **Negative**  **N=345** | **Positive**  **N=45** | **OR** | p.overalla |
| PD_L1 (CPS ≥1) |  |  |  |  | 0.442 |
| Negative | 200 (51.3%) | 174 (50.4%) | 26 (57.8%) | Ref. |  |
| Positive | 190 (48.7%) | 171 (49.6%) | 19 (42.2%) | 0.75 [0.39;1.40] |  |
| PD_L1 (CPS ≥5) |  |  |  |  | 0.088 |
| Negative | 264 (67.7%) | 228 (66.1%) | 36 (80.0%) | Ref. |  |
| Positive | 126 (32.3%) | 117 (33.9%) | 9 (20.0%) | 0.49 [0.22;1.02] |  |
| PD_L1 (CPS ≥10) |  |  |  |  | 0.202 |
| Negative | 325 (83.3%) | 284 (82.3%) | 41 (91.1%) | Ref. |  |
| Positive | 65 (16.7%) | 61 (17.7%) | 4 (8.89%) | 0.47 [0.13;1.23] |  |
| PD_L1 (CPS ≥20) |  |  |  |  | 0.292 |
| Negative | 350 (89.7%) | 307 (89.0%) | 43 (95.6%) | Ref. |  |
| Positive | 40 (10.3%) | 38 (11.0%) | 2 (4.44%) | 0.40 [0.06;1.39] |  |
| **HER2 NGS (copies≥3.0)** | | | | | |
|  | **[ALL]**  **N=390** | **Negative**  **N=348** | **Positive**  **N=42** | **OR** | p.overalla |
| PD_L1 (CPS ≥1) |  |  |  |  | 0.333 |
| Negative | 200 (51.3%) | 175 (50.3%) | 25 (59.5%) | Ref. |  |
| Positive | 190 (48.7%) | 173 (49.7%) | 17 (40.5%) | 0.69 [0.35;1.32] |  |
| PD_L1 (CPS ≥5) |  |  |  |  | 0.077 |
| Negative | 264 (67.7%) | 230 (66.1%) | 34 (81.0%) | Ref. |  |
| Positive | 126 (32.3%) | 118 (33.9%) | 8 (19.0%) | 0.47 [0.19;1.00] |  |
| PD_L1 (CPS ≥10) |  |  |  |  | 0.125 |
| Negative | 325 (83.3%) | 286 (82.2%) | 39 (92.9%) | Ref. |  |
| Positive | 65 (16.7%) | 62 (17.8%) | 3 (7.14%) | 0.37 [0.08;1.07] |  |
| PD_L1 (CPS ≥20) |  |  |  |  | 0.287 |
| Negative | 350 (89.7%) | 310 (89.1%) | 40 (95.2%) | Ref. |  |
| Positive | 40 (10.3%) | 38 (10.9%) | 2 (4.76%) | 0.44 [0.06;1.51] |  |
| **HER2 NGS (copies≥3.5)** | | | | | |
|  | **[ALL]**  **N=390** | **Negative**  **N=349** | **Positive**  **N=41** | **OR** | p.overalla |
| PD_L1 (CPS ≥1) |  |  |  |  | 0.414 |
| Negative | 200 (51.3%) | 176 (50.4%) | 24 (58.5%) | Ref. |  |
| Positive | 190 (48.7%) | 173 (49.6%) | 17 (41.5%) | 0.72 [0.37;1.39] |  |
| PD_L1 (CPS ≥5) |  |  |  |  | 0.094 |
| Negative | 264 (67.7%) | 231 (66.2%) | 33 (80.5%) | Ref. |  |
| Positive | 126 (32.3%) | 118 (33.8%) | 8 (19.5%) | 0.48 [0.20;1.03] |  |
| PD_L1 (CPS ≥10) |  |  |  |  | 0.140 |
| Negative | 325 (83.3%) | 287 (82.2%) | 38 (92.7%) | Ref. |  |
| Positive | 65 (16.7%) | 62 (17.8%) | 3 (7.32%) | 0.38 [0.09;1.11] |  |
| PD_L1 (CPS ≥20) |  |  |  |  | 0.288 |
| Negative | 350 (89.7%) | 311 (89.1%) | 39 (95.1%) | Ref. |  |
| Positive | 40 (10.3%) | 38 (10.9%) | 2 (4.88%) | 0.45 [0.07;1.56] |  |
| **HER2 NGS (copies≥4.0)** | | | | | |
|  | **[ALL]**  **N=390** | **Negative**  **N=366** | **Positive**  **N=24** | **OR** | p.overalla |
| PD_L1 (CPS ≥1) |  |  |  |  | 0.615 |
| Negative | 200 (51.3%) | 186 (50.8%) | 14 (58.3%) | Ref. |  |
| Positive | 190 (48.7%) | 180 (49.2%) | 10 (41.7%) | 0.74 [0.31;1.72] |  |
| PD_L1 (CPS ≥5) |  |  |  |  | 0.310 |
| Negative | 264 (67.7%) | 245 (66.9%) | 19 (79.2%) | Ref. |  |
| Positive | 126 (32.3%) | 121 (33.1%) | 5 (20.8%) | 0.55 [0.17;1.41] |  |
| PD_L1 (CPS ≥10) |  |  |  |  | 0.396 |
| Negative | 325 (83.3%) | 303 (82.8%) | 22 (91.7%) | Ref. |  |
| Positive | 65 (16.7%) | 63 (17.2%) | 2 (8.33%) | 0.47 [0.07;1.65] |  |
| PD_L1 (CPS ≥20) |  |  |  |  | 0.492 |
| Negative | 350 (89.7%) | 327 (89.3%) | 23 (95.8%) | Ref. |  |
| Positive | 40 (10.3%) | 39 (10.7%) | 1 (4.17%) | 0.41 [0.02;2.05] |  |
| **HER2 NGS (copies≥5.0)** | | | | | |
|  | **[ALL]**  **N=390** | **Negative**  **N=372** | **Positive**  **N=18** | **OR** | p.overalla |
| PD_L1 (CPS ≥1) |  |  |  |  | 0.897 |
| Negative | 200 (51.3%) | 190 (51.1%) | 10 (55.6%) | Ref. |  |
| Positive | 190 (48.7%) | 182 (48.9%) | 8 (44.4%) | 0.84 [0.31;2.20] |  |
| PD_L1 (CPS ≥5) |  |  |  |  | 0.497 |
| Negative | 264 (67.7%) | 250 (67.2%) | 14 (77.8%) | Ref. |  |
| Positive | 126 (32.3%) | 122 (32.8%) | 4 (22.2%) | 0.60 [0.16;1.74] |  |
| PD_L1 (CPS ≥10) |  |  |  |  | 0.330 |
| Negative | 325 (83.3%) | 308 (82.8%) | 17 (94.4%) | Ref. |  |
| Positive | 65 (16.7%) | 64 (17.2%) | 1 (5.56%) | 0.32 [0.01;1.61] |  |
| PD_L1 (CPS ≥20) |  |  |  |  | 1.000 |
| Negative | 350 (89.7%) | 333 (89.5%) | 17 (94.4%) | Ref. |  |
| Positive | 40 (10.3%) | 39 (10.5%) | 1 (5.56%) | 0.57 [0.02;2.90] |  |
| **HER2 NGS (copies≥6.0)** | | | | | |
|  | **[ALL]**  **N=390** | **Negative**  **N=378** | **Positive**  **N=12** | **OR** | p.overalla |
| PD_L1 (CPS ≥1) |  |  |  |  | 1.000 |
| Negative | 200 (51.3%) | 194 (51.3%) | 6 (50.0%) | Ref. |  |
| Positive | 190 (48.7%) | 184 (48.7%) | 6 (50.0%) | 1.05 [0.32;3.51] |  |
| PD_L1 (CPS ≥5) |  |  |  |  | 0.352 |
| Negative | 264 (67.7%) | 254 (67.2%) | 10 (83.3%) | Ref. |  |
| Positive | 126 (32.3%) | 124 (32.8%) | 2 (16.7%) | 0.43 [0.06;1.71] |  |
| PD_L1 (CPS ≥10) |  |  |  |  | 0.230 |
| Negative | 325 (83.3%) | 313 (82.8%) | 12 (100%) | Ref. |  |
| Positive | 65 (16.7%) | 65 (17.2%) | 0 (0.00%) | [.;.] |  |
| PD_L1 (CPS ≥20) |  |  |  |  | 0.621 |
| Negative | 350 (89.7%) | 338 (89.4%) | 12 (100%) | Ref. |  |
| Positive | 40 (10.3%) | 40 (10.6%) | 0 (0.00%) | [.;.] |  |

**Table S6 Summary of frequency of tumor signaling pathway alterations grouped by PD-L1strata(CPS≥1).**

| **Pathway** | **[ALL]**  **N=390** | **Negative**  **N=200** | **Positive**  **N=190** | **OR** | p**^a^** | p.adj**^b^** | **p.signif** |
| --- | --- | --- | --- | --- | --- | --- | --- |
| Cell cycle | 1.67 (4.62) | 1.44 (4.00) | 1.91 (5.19) | 1.02 [0.98;1.07] | 0.569368602 | 0.57 | ns |
| Chromatin histone modifiers | 1.86 (4.28) | 1.47 (3.61) | 2.28 (4.87) | 1.05 [1.00;1.10] | 0.146927796 | 0.15 | ns |
| Chromatin other | 0.71 (2.64) | 0.36 (1.86) | 1.09 (3.23) | 1.13 [1.03;1.23] | 0.004912381 | 0.0049 | ** |
| Chromatin SWI_SNF complex | 3.08 (6.90) | 2.69 (5.73) | 3.49 (7.94) | 1.02 [0.99;1.05] | 0.754332698 | 0.75 | ns |
| Epigenetics DNA modifiers | 1.03 (10.1) | 0.50 (7.07) | 1.58 (12.5) | 1.01 [0.99;1.03] | 0.292280662 | 0.29 | ns |
| Genome integrity | 5.84 (5.22) | 5.39 (4.94) | 6.32 (5.49) | 1.04 [1.00;1.08] | 0.091469315 | 0.091 | ns |
| Histone modification | 1.20 (6.21) | 1.00 (5.70) | 1.40 (6.71) | 1.01 [0.98;1.04] | 0.522117247 | 0.52 | ns |
| Immune signaling | 0.08 (0.87) | 0.10 (1.00) | 0.05 (0.73) | 0.94 [0.74;1.19] | 0.595058221 | 0.6 | ns |
| MAPK signaling | 1.99 (5.33) | 1.83 (4.42) | 2.16 (6.14) | 1.01 [0.97;1.05] | 0.814692805 | 0.81 | ns |
| Metabolism | 0.77 (6.16) | 0.75 (6.09) | 0.79 (6.25) | 1.00 [0.97;1.03] | 0.951257318 | 0.95 | ns |
| NFKβ signaling | 0.64 (5.63) | 0.25 (3.54) | 1.05 (7.20) | 1.03 [0.99;1.08] | 0.160205442 | 0.16 | ns |
| NOTCH signaling | 2.82 (16.6) | 2.00 (14.0) | 3.68 (18.9) | 1.01 [0.99;1.02] | 0.316677674 | 0.32 | ns |
| Other | 0.33 (1.22) | 0.43 (1.41) | 0.22 (0.97) | 0.86 [0.72;1.02] | 0.096182112 | 0.096 | ns |
| Other signaling | 1.18 (2.10) | 1.18 (2.18) | 1.18 (2.01) | 1.00 [0.91;1.10] | 0.821072622 | 0.82 | ns |
| PI3K signaling | 2.71 (6.06) | 2.28 (5.49) | 3.16 (6.60) | 1.02 [0.99;1.06] | 0.185609006 | 0.19 | ns |
| Protein homeostasis_ubiquitination | 0.67 (2.17) | 0.40 (1.72) | 0.95 (2.53) | 1.13 [1.02;1.25] | 0.009225711 | 0.0092 | ** |
| RNA abundance | 0.31 (1.40) | 0.23 (1.23) | 0.39 (1.56) | 1.08 [0.94;1.25] | 0.282611239 | 0.28 | ns |
| RTK signaling | 3.70 (5.40) | 3.91 (5.51) | 3.49 (5.28) | 0.99 [0.95;1.02] | 0.416360468 | 0.42 | ns |
| Splicing | 0.51 (3.12) | 0.42 (3.10) | 0.61 (3.15) | 1.02 [0.96;1.09] | 0.322024539 | 0.32 | ns |
| TGFβ signaling | 1.39 (4.48) | 1.14 (4.14) | 1.65 (4.81) | 1.03 [0.98;1.07] | 0.228833144 | 0.23 | ns |
| TOR signaling | 1.62 (7.19) | 1.50 (6.93) | 1.75 (7.46) | 1.00 [0.98;1.03] | 0.727636805 | 0.73 | ns |
| Transcription factor | 0.39 (1.07) | 0.38 (1.05) | 0.39 (1.10) | 1.01 [0.84;1.21] | 0.974861262 | 0.97 | ns |
| Wnt_β_catenin signaling | 3.40 (7.95) | 2.88 (7.59) | 3.95 (8.29) | 1.02 [0.99;1.04] | 0.166077611 | 0.17 | ns |

^a^P value are tested by Wilcoxon Test; ^b^P value adjustment are tested by Benjamini-Hochberg-adjusted Test

**Table S7 Summary of frequency of tumor signaling pathway alterations grouped by PD-L1strata(CPS≥5).**

| **Pathway** | **[ALL]**  **N=390** | **Negative**  **N=264** | **Positive**  **N=126** | **OR** | **p^a^** | **p.adj^b^** | **p.signif** |
| --- | --- | --- | --- | --- | --- | --- | --- |
| Cell cycle | 1.67 (4.62) | 1.75 (4.86) | 1.49 (4.06) | 0.99 [0.94;1.04] | 0.828387012 | 0.83 | ns |
| Chromatin histone modifiers | 1.86 (4.28) | 1.52 (3.86) | 2.59 (4.99) | 1.06 [1.01;1.11] | 0.021304375 | 0.021 | * |
| Chromatin other | 0.71 (2.64) | 0.46 (2.06) | 1.25 (3.51) | 1.11 [1.03;1.20] | 0.009472788 | 0.0095 | ** |
| Chromatin SWI_SNF complex | 3.08 (6.90) | 2.46 (5.55) | 4.37 (8.99) | 1.04 [1.01;1.07] | 0.104871587 | 0.1 | ns |
| Epigenetics DNA modifiers | 1.03 (10.1) | 0.76 (8.69) | 1.59 (12.5) | 1.01 [0.99;1.03] | 0.449153522 | 0.45 | ns |
| Genome integrity | 5.84 (5.22) | 5.65 (4.82) | 6.24 (5.99) | 1.02 [0.98;1.06] | 0.561345277 | 0.56 | ns |
| Histone modification | 1.20 (6.21) | 0.76 (4.98) | 2.12 (8.16) | 1.03 [1.00;1.07] | 0.043423178 | 0.043 | * |
| Immune signaling | 0.08 (0.87) | 0.08 (0.87) | 0.08 (0.89) | 1.00 [0.79;1.28] | 0.972150591 | 0.97 | ns |
| MAPK signaling | 1.99 (5.33) | 1.73 (4.37) | 2.56 (6.91) | 1.03 [0.99;1.07] | 0.604912108 | 0.6 | ns |
| Metabolism | 0.77 (6.16) | 0.76 (6.12) | 0.79 (6.27) | 1.00 [0.97;1.04] | 0.958674329 | 0.96 | ns |
| NFKβ signaling | 0.64 (5.63) | 0.57 (5.31) | 0.79 (6.27) | 1.01 [0.97;1.04] | 0.713434652 | 0.71 | ns |
| NOTCH signaling | 2.82 (16.6) | 2.65 (16.1) | 3.17 (17.6) | 1.00 [0.99;1.01] | 0.772010424 | 0.77 | ns |
| Other | 0.33 (1.22) | 0.34 (1.27) | 0.29 (1.11) | 0.96 [0.80;1.15] | 0.752265676 | 0.75 | ns |
| Other signaling | 1.18 (2.10) | 1.15 (2.11) | 1.25 (2.09) | 1.02 [0.92;1.13] | 0.617056838 | 0.62 | ns |
| PI3K signaling | 2.71 (6.06) | 2.23 (5.66) | 3.70 (6.74) | 1.04 [1.00;1.07] | 0.010581604 | 0.011 | * |
| Protein homeostasis_ubiquitination | 0.67 (2.17) | 0.48 (1.82) | 1.06 (2.72) | 1.12 [1.02;1.23] | 0.016623565 | 0.017 | * |
| RNA abundance | 0.31 (1.40) | 0.23 (1.21) | 0.48 (1.72) | 1.12 [0.97;1.30] | 0.100999062 | 0.1 | ns |
| RTK signaling | 3.70 (5.40) | 3.86 (5.48) | 3.37 (5.22) | 0.98 [0.94;1.02] | 0.330033264 | 0.33 | ns |
| Splicing | 0.51 (3.12) | 0.38 (2.88) | 0.79 (3.56) | 1.04 [0.98;1.11] | 0.112736226 | 0.11 | ns |
| TGFβ signaling | 1.39 (4.48) | 1.08 (3.99) | 2.04 (5.33) | 1.05 [1.00;1.09] | 0.044816413 | 0.045 | * |
| TOR signaling | 1.62 (7.19) | 1.89 (7.73) | 1.06 (5.87) | 0.98 [0.95;1.02] | 0.283305382 | 0.28 | ns |
| Transcription factor | 0.39 (1.07) | 0.36 (1.00) | 0.45 (1.22) | 1.08 [0.89;1.30] | 0.608229516 | 0.61 | ns |
| Wnt_β_catenin signaling | 3.40 (7.95) | 2.70 (7.18) | 4.86 (9.22) | 1.03 [1.01;1.06] | 0.013747448 | 0.014 | * |

^a^P value are tested by Wilcoxon Test; ^b^P value adjustment are tested by Benjamini-Hochberg-adjusted Test

**Table S8 Summary of frequency of tumor signaling pathway alterations grouped by PD-L1strata(CPS≥10).**

| **Pathway** | **[ALL]**  **N=390** | **Negative**  **N=325** | **Positive**  **N=65** | **OR** | p**^a^** | p.adj**^b^** | **p.signif** |
| --- | --- | --- | --- | --- | --- | --- | --- |
| Cell cycle | 1.67 (4.62) | 1.58 (4.60) | 2.12 (4.72) | 1.02 [0.97;1.08] | 0.233764278 | 0.23 | ns |
| Chromatin histone modifiers | 1.86 (4.28) | 1.58 (3.90) | 3.28 (5.67) | 1.08 [1.02;1.13] | 0.006061504 | 0.0061 | ** |
| Chromatin other | 0.71 (2.64) | 0.59 (2.34) | 1.32 (3.77) | 1.09 [1.00;1.18] | 0.112275981 | 0.11 | ns |
| Chromatin SWI_SNF complex | 3.08 (6.90) | 2.73 (6.19) | 4.81 (9.55) | 1.04 [1.00;1.07] | 0.181350607 | 0.18 | ns |
| Epigenetics DNA modifiers | 1.03 (10.1) | 1.23 (11.0) | 0.00 (0.00) | 0.87 [0.00;1.35] | 0.371085037 | 0.37 | ns |
| Genome integrity | 5.84 (5.22) | 5.91 (5.12) | 5.49 (5.75) | 0.98 [0.93;1.04] | 0.325793651 | 0.33 | ns |
| Histone modification | 1.20 (6.21) | 0.92 (5.48) | 2.56 (8.95) | 1.03 [1.00;1.07] | 0.051983056 | 0.052 | ns |
| Immune signaling | 0.08 (0.87) | 0.06 (0.78) | 0.15 (1.24) | 1.10 [0.86;1.40] | 0.439751395 | 0.44 | ns |
| MAPK signaling | 1.99 (5.33) | 1.78 (4.85) | 3.08 (7.22) | 1.04 [1.00;1.08] | 0.287730894 | 0.29 | ns |
| Metabolism | 0.77 (6.16) | 0.77 (6.16) | 0.77 (6.20) | 1.00 [0.96;1.04] | 1 | 1 | ns |
| NFKβ signaling | 0.64 (5.63) | 0.62 (5.52) | 0.77 (6.20) | 1.00 [0.96;1.05] | 0.843089339 | 0.84 | ns |
| NOTCH signaling | 2.82 (16.6) | 2.77 (16.4) | 3.08 (17.4) | 1.00 [0.99;1.02] | 0.893002758 | 0.89 | ns |
| Other | 0.33 (1.22) | 0.31 (1.20) | 0.42 (1.33) | 1.07 [0.88;1.31] | 0.428250055 | 0.43 | ns |
| Other signaling | 1.18 (2.10) | 1.14 (2.09) | 1.37 (2.16) | 1.05 [0.93;1.18] | 0.365671755 | 0.37 | ns |
| PI3K signaling | 2.71 (6.06) | 2.46 (5.92) | 3.93 (6.64) | 1.04 [1.00;1.08] | 0.031570321 | 0.032 | * |
| Protein homeostasis_ubiquitination | 0.67 (2.17) | 0.51 (1.93) | 1.44 (3.00) | 1.16 [1.05;1.28] | 0.001054296 | 0.0011 | ** |
| RNA abundance | 0.31 (1.40) | 0.27 (1.31) | 0.51 (1.79) | 1.11 [0.95;1.30] | 0.196415188 | 0.2 | ns |
| RTK signaling | 3.70 (5.40) | 3.67 (5.30) | 3.85 (5.92) | 1.01 [0.96;1.06] | 0.857908347 | 0.86 | ns |
| Splicing | 0.51 (3.12) | 0.36 (2.75) | 1.28 (4.48) | 1.07 [1.00;1.14] | 0.009799045 | 0.0098 | ** |
| TGFβ signaling | 1.39 (4.48) | 1.32 (4.29) | 1.76 (5.36) | 1.02 [0.97;1.08] | 0.621361199 | 0.62 | ns |
| TOR signaling | 1.62 (7.19) | 1.74 (7.43) | 1.03 (5.80) | 0.98 [0.94;1.03] | 0.463080083 | 0.46 | ns |
| Transcription factor | 0.39 (1.07) | 0.40 (1.09) | 0.32 (0.96) | 0.92 [0.70;1.21] | 0.547859384 | 0.55 | ns |
| Wnt_β_catenin signaling | 3.40 (7.95) | 2.92 (7.29) | 5.77 (10.4) | 1.04 [1.01;1.07] | 0.03418087 | 0.034 | * |

^a^P value are tested by Wilcoxon Test; ^b^P value adjustment are tested by Benjamini-Hochberg-adjusted Test

**Table S9 Summary of frequency of tumor signaling pathway alterations grouped by PD-L1strata(CPS≥20).**

| **Pathway** | **[ALL]**  **N=390** | **Negative**  **N=350** | **Positive**  **N=40** | **OR** | **p^a^** | **p.adj^b^** | **p.signif** |
| --- | --- | --- | --- | --- | --- | --- | --- |
| Cell cycle | 1.67 (4.62) | 1.61 (4.60) | 2.19 (4.81) | 1.02 [0.96;1.09] | 0.309910023 | 0.31 | ns |
| Chromatin histone modifiers | 1.86 (4.28) | 1.70 (4.14) | 3.33 (5.23) | 1.07 [1.01;1.13] | 0.012709086 | 0.013 | * |
| Chromatin other | 0.71 (2.64) | 0.59 (2.38) | 1.79 (4.20) | 1.12 [1.03;1.22] | 0.012627182 | 0.013 | * |
| Chromatin SWI_SNF complex | 3.08 (6.90) | 2.86 (6.48) | 5.00 (9.72) | 1.04 [1.00;1.08] | 0.261848525 | 0.26 | ns |
| Epigenetics DNA modifiers | 1.03 (10.1) | 1.14 (10.6) | 0.00 (0.00) | 0.87 [0.00;1.36] | 0.49999121 | 0.5 | ns |
| Genome integrity | 5.84 (5.22) | 5.84 (5.11) | 5.89 (6.24) | 1.00 [0.94;1.07] | 0.679719038 | 0.68 | ns |
| Histone modification | 1.20 (6.21) | 1.05 (5.82) | 2.50 (8.89) | 1.03 [0.99;1.07] | 0.161762348 | 0.16 | ns |
| Immune signaling | 0.08 (0.87) | 0.06 (0.75) | 0.25 (1.58) | 1.16 [0.91;1.48] | 0.188183131 | 0.19 | ns |
| MAPK signaling | 1.99 (5.33) | 1.94 (5.27) | 2.50 (5.89) | 1.02 [0.96;1.07] | 0.582850265 | 0.58 | ns |
| Metabolism | 0.77 (6.16) | 0.71 (5.94) | 1.25 (7.91) | 1.01 [0.97;1.06] | 0.604852522 | 0.6 | ns |
| NFKβ signaling | 0.64 (5.63) | 0.57 (5.32) | 1.25 (7.91) | 1.02 [0.97;1.06] | 0.472723875 | 0.47 | ns |
| NOTCH signaling | 2.82 (16.6) | 2.57 (15.9) | 5.00 (22.1) | 1.01 [0.99;1.02] | 0.381479099 | 0.38 | ns |
| Other | 0.33 (1.22) | 0.31 (1.20) | 0.45 (1.38) | 1.09 [0.86;1.37] | 0.423882258 | 0.42 | ns |
| Other signaling | 1.18 (2.10) | 1.15 (2.06) | 1.43 (2.40) | 1.06 [0.92;1.22] | 0.607587145 | 0.61 | ns |
| PI3K signaling | 2.71 (6.06) | 2.57 (6.07) | 3.89 (5.93) | 1.03 [0.98;1.08] | 0.041782716 | 0.042 | * |
| Protein homeostasis_ubiquitination | 0.67 (2.17) | 0.59 (2.09) | 1.33 (2.70) | 1.12 [1.00;1.26] | 0.01457101 | 0.015 | * |
| RNA abundance | 0.31 (1.40) | 0.27 (1.31) | 0.67 (2.03) | 1.16 [0.97;1.38] | 0.087434215 | 0.087 | ns |
| RTK signaling | 3.70 (5.40) | 3.66 (5.28) | 4.06 (6.42) | 1.01 [0.96;1.07] | 0.882320968 | 0.88 | ns |
| Splicing | 0.51 (3.12) | 0.33 (2.66) | 2.08 (5.58) | 1.10 [1.03;1.18] | 0.000103325 | 0.0001 | *** |
| TGFβ signaling | 1.39 (4.48) | 1.39 (4.37) | 1.43 (5.41) | 1.00 [0.93;1.08] | 0.724226021 | 0.72 | ns |
| TOR signaling | 1.62 (7.19) | 1.71 (7.37) | 0.83 (5.27) | 0.98 [0.92;1.04] | 0.463795763 | 0.46 | ns |
| Transcription factor | 0.39 (1.07) | 0.40 (1.08) | 0.26 (0.97) | 0.86 [0.59;1.25] | 0.287487556 | 0.29 | ns |
| Wnt_β_catenin signaling | 3.40 (7.95) | 3.18 (7.64) | 5.31 (10.2) | 1.03 [0.99;1.06] | 0.246640205 | 0.25 | ns |

^a^P value are tested by Wilcoxon Test; ^b^P value adjustment are tested by Benjamini-Hochberg-adjusted Test
